# A nematode-trapping fungus orchestrates polarity cues, septins, and NOX signaling for trap formation

**DOI:** 10.1083/jcb.202603155

**Published:** 2026-10-19

**Authors:** Chih-Yen Kuo, Hung-Che Lin, Yun-Zhen Chou, Sheng-An Chen, Hillel Schwartz, Yen-Ping Hsueh

**Affiliations:** 1 Department of Complex Biological Interactions, Max Planck Institute for Biology, Tübingen, Germany; 2 Institute of Molecular Biology, Academia Sinica, Taipei, Taiwan

## Abstract

Reprogramming cell morphogenesis in response to environmental cues is fundamental to microbial adaptation and survival. The nematode-trapping fungus (NTF) *Arthrobotrys oligospora* exemplifies this plasticity by developing adhesive traps to capture nematodes. However, mechanisms underlying trap morphogenesis and host invasion remain unclear. Using live-cell imaging, genetics, and functional assays, we show trap morphogenesis proceeds through localized redirection of hyphal growth followed by site-specific hyphal fusion, whereas infection development involves a transition from isotropic infection bulb expansion to multipolar invasive growth. These transitions are coordinated by the reorganization of conserved polarity proteins, asymmetric septin localization at the trap cell inner rim, and actin remodeling. Nematode-induced Nox1 signaling is required to recruit polarity and cytoskeletal machinery to the trap cell fusion site. Together, these findings establish NTF as a powerful model for understanding how conserved morphogenetic machinery drives the transition from linear hyphae to hyphal loops to trap nematodes.

## Teaser

Kuo et al. demonstrate that dynamic reorganization of polarity proteins, septins, and actin, together with Nox1-dependent ROS signaling, drives trap morphogenesis and cell–cell fusion in *Arthrobotrys oligospora*. This work reveals conserved mechanisms that enable fungi to remodel hyphal architecture for nematode predation and host invasion.

## Introduction

Fungal survival and pathogenicity depend on the ability to remodel cellular architecture in response to environmental cues (Riquelme et al., 2018). This morphogenetic plasticity enables fungi to switch between growth forms or develop specialized infection structures, such as host-penetrating appressoria (Boyce and Andrianopoulos, 2015; Ryder and Talbot, 2015). Cell polarity is the primary organizer of these transitions (Asnacios and Hamant, 2012; Chiou et al., 2017). In the fission yeast *Schizosaccharomyces pombe,* the cell-end markers Tea1 and Tea4 are delivered by microtubules and anchored by Mod5 to define sites of polarized growth (Martin et al., 2005; Mata and Nurse, 1997; Snaith and Sawin, 2003). In filamentous fungi, this conserved polarity machinery supports continuous apical hyphal extension (Kriegler et al., 2025a; Takeshita et al., 2013). During hyphal extension, chitin synthases (CHSs) are delivered along actin cables to the Spitzenkörper (SPK), where polarized secretion directs localized chitin deposition at the hyphal tip (Riquelme and Sánchez-León, 2014). While linear growth relies on steady apical extension, complex morphogenesis requires dynamic reorganization of polarity cues and membrane architecture.

The cytoskeleton acts as both a transport track and a scaffold for shape remodeling (Riquelme et al., 2018). Actin filaments form apical patches and contractile rings to mediate endocytosis and septum formation (Berepiki et al., 2010; Berepiki et al., 2011). Septins are a conserved family of GTP-binding proteins that assemble into filaments and ringlike structures and are often regarded as a fourth cytoskeletal element in animals and fungi (Mostowy and Cossart, 2012). They preferentially assemble at regions of micron-scale membrane curvature, such as yeast mother–bud necks and hyphal branches (Bridges et al., 2016; Khan et al., 2015). In the rice blast fungus *Magnaporthe oryzae*, septins assemble at the appressorium pore to recruit a toroidal F-actin network, which is anchored to the plasma membrane via phosphoinositide interactions and stabilized by Tea1 (Dagdas et al., 2012). These examples highlight the tight coordination between cell polarity proteins and cytoskeletal dynamics in fungal morphogenesis.

Cell fusion is a fundamental process in fungal growth and development, enabling the establishment of interconnected hyphal networks (Fischer and Glass, 2019). Best characterized during the formation of conidial anastomosis tubes, this process involves MAP kinase signaling that activates NADPH oxidases (NOX enzymes) (Herzog et al., 2015). These enzymes generate reactive oxygen species (ROS) that act as localized signals to trigger cell wall remodeling and membrane merger at the fusion interface (Read et al., 2012; Roca et al., 2012). NOX-derived ROS serve as essential regulators of fungal development, controlling processes from hyphal branching to sexual reproduction (Tudzynski et al., 2012).

Nematode-trapping fungi (NTF) represent a powerful model for studying fungal morphogenetic plasticity. Under nutrient-limiting conditions, NTF sense nematode-derived signals and shift from a saprophytic to a predatory lifestyle by developing trapping structures to capture nematodes as a nutrient source (Nordbring-Hertz et al., 2001). In response to prey, the NTF *Arthrobotrys oligospora* produces adhesive traps through a stereotypic program where specialized hyphae grow, curve, and fuse to form closed loops that generate intricate three-dimensional networks. Signaling pathways governing trap induction have been characterized (Chen et al., 2021; Chen et al., 2022; Hu et al., 2024; Kuo et al., 2024). In addition, several cellular regulators required for trap development have been identified in NTF, including cell-end markers, septins, and NADPH oxidase signaling (Kriegler et al., 2025b; Kriegler et al., 2026; Li et al., 2017; Zhu et al., 2025). However, how cell polarity proteins, the cytoskeleton, and ROS signaling are dynamically coordinated to drive trap morphogenesis and subsequent penetration to nematodes remains largely unclear.

In this study, we asked how *A*. *oligospora* reprograms conserved cellular machinery to facilitate the transition from a saprophytic to a predatory lifestyle. Using live-cell imaging, quantitative image analysis, and functional genetics, we show that cell polarity proteins and cytoskeletal systems are dynamically reorganized during trap morphogenesis and prey penetration. Specifically, we demonstrate that trap morphogenesis proceeds through localized redirection of hyphal growth followed by site-specific hyphal fusion. During this process, cell-end markers and CHSs are dynamically reorganized, septins transiently accumulate along the inner rim of developing loops as curvature forms, and localized Nox1-mediated ROS signaling is required for recruitment of the polarity factors and cell–cell fusion. We further demonstrate that prey penetration and invasion involves septin ring assembly in the infection bulb and the infection bulb transitions from isotropic growth to multipolar growth to generate invasive hyphae. Together, our study reveals how cell polarity proteins and cytoskeletal regulators coordinate morphogenetic transitions in NTF, providing new insights into the fundamental mechanisms of polarized growth and network assembly in filamentous fungi.

## Results

### Identification and characterization of CHSs and cell-end markers in *A. oligospora*

**Figure S1.**
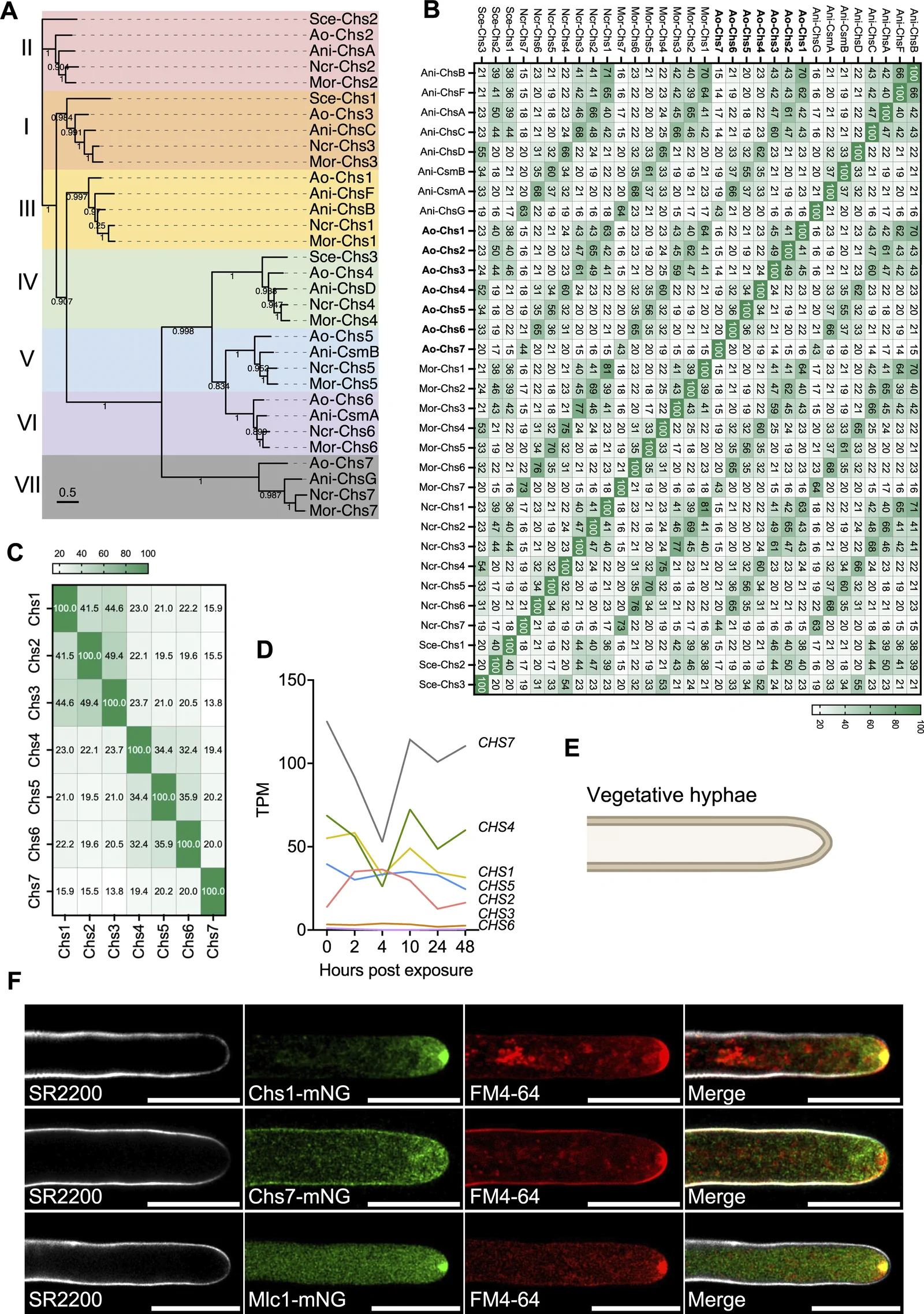
**CHSs are evolutionarily conserved and localize to the vegetative hyphal apex in *A. oligospora*. (A)** Maximum-likelihood phylogeny of fungal CHSs. Roman numerals on the left denote the CHS classes. Node numbers indicate support values ranging from 0 to 1. The scale bar represents 0.5–amino acid substitutions per site. Species abbreviations: Ani, *A. nidulans*; Ao, *A. oligospora*; Mor, *M. oryzae*; Ncr, *N. crassa*; Sce, *S. cerevisiae*. **(B and C)** Pairwise amino acid sequence identity among fungal CHSs (B) and *A. oligospora* CHSs (C). The color gradient signifies the degree of conservation, where darker shading corresponds to higher identity percentages. Numbers indicate sequence identity percentages. Species abbreviations are as defined in A. **(D)** TPM values of the differentially expressed *CHS*s in a published RNA-seq time course following exposure of *A. oligospora* to *C. elegans* (Lin et al., 2023). Values represent the mean of three biological replicates. **(E)** Schematic representation of a vegetative hyphal apex. **(F)** Localization of Chs1-mNG, Chs7-mNG, and Mlc1-mNG at the vegetative hyphal apex. Merged images show mNG fluorescence, SR2200-stained cell walls, and FM4-64–labeled membranes and the SPK. Scale bars, 10 μm. Images represent three independent biological replicates. TPM, transcripts per kilobase million.

To investigate the molecular basis of polarized growth and trap morphogenesis, we identified gene families involved in chitin synthesis and cell polarity in *A. oligospora*. Seven CHS genes were identified in the genome of strain TWF154 (Table S1) (Yang et al., 2020), representing the seven conserved classes found in filamentous fungi (Fig. S1 A). Chs1 to Chs6 share ∼60% identity with their respective orthologs and exhibit significant internal divergence (Fig. S1 B). For example, Chs1-Chs3 exhibit roughly 45% pairwise identity, while Chs4 to Chs6 share ∼35% identity. Chs7 is the most divergent member, displaying only 15–20% identity to other CHSs (Fig. S1 B).

To identify which CHSs are relevant to trap morphogenesis, we examined published *A. oligospora* time course RNA-seq data following *Caenorhabditis elegans* exposure (Lin et al., 2023). Transcriptional profiling identified *CHS1*, *CHS4*, and *CHS7* were transiently downregulated at 4 h after exposure (hpe) and recovered by 10 hpe during trap formation (Fig. S1 D). Despite their shared expression profile, these three proteins are highly divergent from each other, sharing only about 20% pairwise identity (Fig. S1 C).

To examine subcellular localization of CHS in vegetative hyphae (Fig. S1 E), Chs1 and Chs7 were C-terminally tagged with mNeonGreen (mNG) under their endogenous promoters, and the myosin light chain Mlc1 was also fused to mNG to serve as a marker for the SPK (Crampin et al., 2005; Giraldo et al., 2013). As expected, Mlc1-mNG localized as a bright apical punctum corresponding to the FM4-64–labeled SPK at the hyphal tip (Fig. S1 F). We observed that Chs1-mNG concentrated as a sharp central punctum that overlapped with the FM4-64–labeled SPK, whereas Chs7-mNG displayed a diffuse apical distribution (Fig. S1 F), suggesting the two CHS might play different roles at the hyphal tip.

**Figure S2.**
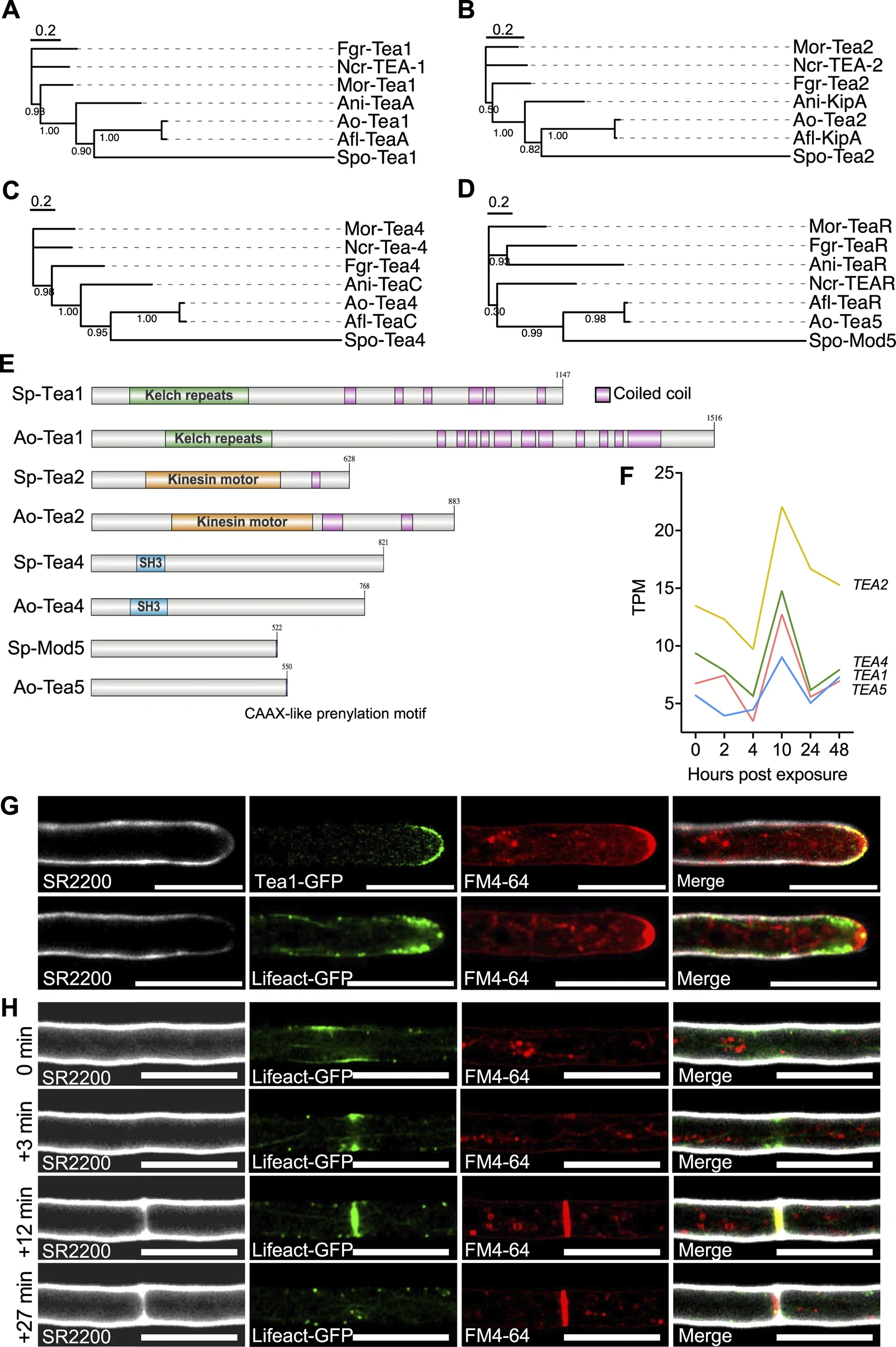
**Cell-end markers are evolutionarily conserved, and Tea1 and actin exhibit polarized localization in *A. oligospora*. (A–D)** Maximum-likelihood phylogenies of Tea1 (A), Tea2 (B), Tea4 (C), and Tea5 (D) proteins from representative fungi. Node numbers indicate support values ranging from 0 to 1. The scale bar represents 0.2–amino acid substitutions per site Species abbreviations: Ani, *A. nidulans*; Ao, *A. oligospora*; Fgr, *F. graminearum*; Mor, *M. oryzae*; Ncr, *N. crassa*; Sce, *S. cerevisiae*; Spo, *S. pombe*. **(E)** Domain architecture of Tea1, Tea2, Tea4, Mod5, and Tea5 proteins from *S. pombe* and *A. oligospora*. Tea1 contains kelch repeat and C-terminal coiled-coil domains, Tea2 contains a kinesin motor and coiled-coil domains, Tea4 contains an SH3 domain, and Mod5 and Tea5 contain a C-terminal CAAX-like prenylation motif. **(F)** TPM values of differentially expressed cell-end marker genes in a published RNA-seq time course following exposure of *A. oligospora* to *C. elegans* (Lin et al., 2023). Values represent the mean of three biological replicates. **(G)** Localization of Tea1-GFP and Lifeact-GFP at the vegetative hyphal apex. **(H)** F-actin localizes to the prospective septation site during septum formation. Hyphae were stained with SR2200 to visualize cell walls and FM4-64 to label the plasma membrane and the SPK. For G and H, merged images show SR2200, GFP, and FM4-64 fluorescence. Scale bars, 10 μm. Images are representative of three independent biological replicates. TPM, transcripts per kilobase million.

To assess whether conserved fungal cell polarity machinery contributes to trap morphogenesis, we searched the *A. oligospora* genome for homologs of the *S. pombe* cell-end markers. We could identify orthologs of Tea1, Tea2, Tea4, and the membrane anchor Mod5, which we renamed Tea5 in *A. oligospora*, but found no evidence of the presence of a Tea3 homolog (Table S1). Phylogenetic analysis demonstrated that these proteins are most closely related to orthologs in *Aspergillus nidulans* and the closely related NTF *Arthrobotrys flagrans* (Fig. S2, A–D). Domain architecture analysis revealed high conservation of functional motifs, including kelch repeats in Tea1, a kinesin motor domain in Tea2, an SH3 domain in Tea4, and a C-terminal CAAX-like prenylation motif in Tea5 (Fig. S2 E).

RNA-seq analysis showed that *TEA1*, *TEA2*, *TEA4*, and *TEA5* are nematode-responsive genes, with peak expression at 10 hpe (Fig. S2 F). To examine Tea1 subcellular localization, we generated a strain expressing Tea1-GFP fusion under its native promoter. Tea1-GFP localized predominantly to the peripheral cortex of the hyphal apex, in contrast to the SPK localization of Chs1 (Fig. S1 F and Fig. S2 G).

### Cytoskeletal networks display distinct localization patterns at the tip and curvature of vegetative hyphae

Actin and septins are cytoskeleton proteins that play essential roles in fungal cell polarity and morphogenesis (Riquelme et al., 2018). To visualize F-actin organization, Lifeact-GFP was expressed under the constitutive promoter in *A. oligospora*. During hyphal extension, Lifeact-GFP localized to the SPK and peripheral actin patches at the hyphal apex (Fig. S2 G and Video 1). During septation, a medial actin ring assembled before membrane ingression, as revealed by FM4-64 staining, and constricted during septum formation, and gradually diminished upon completion of cytokinesis (Fig. S2 H and Video 1). These dynamics indicate that actin ring assembly and constriction are closely associated with septum formation during cytokinesis.

**Video 1.** **Dynamic reorganization of F-actin during hyphal extension and septum formation.** Time-lapse fluorescence imaging of an *A. oligospora* hypha expressing Lifeact-GFP during vegetative hyphal growth and septum formation. Hyphae were costained with SR2200 to label cell walls (white) and FM4-64 to visualize the plasma membrane and the SPK (red). This video corresponds to the micrographs shown in Fig. S2, G and H. Images were acquired every 1.5 min and are displayed at 2 frames per second. The timestamp indicates the time elapsed and is displayed in HH:MM:SS format. Scale bar, 10 μm.

**Figure S3.**
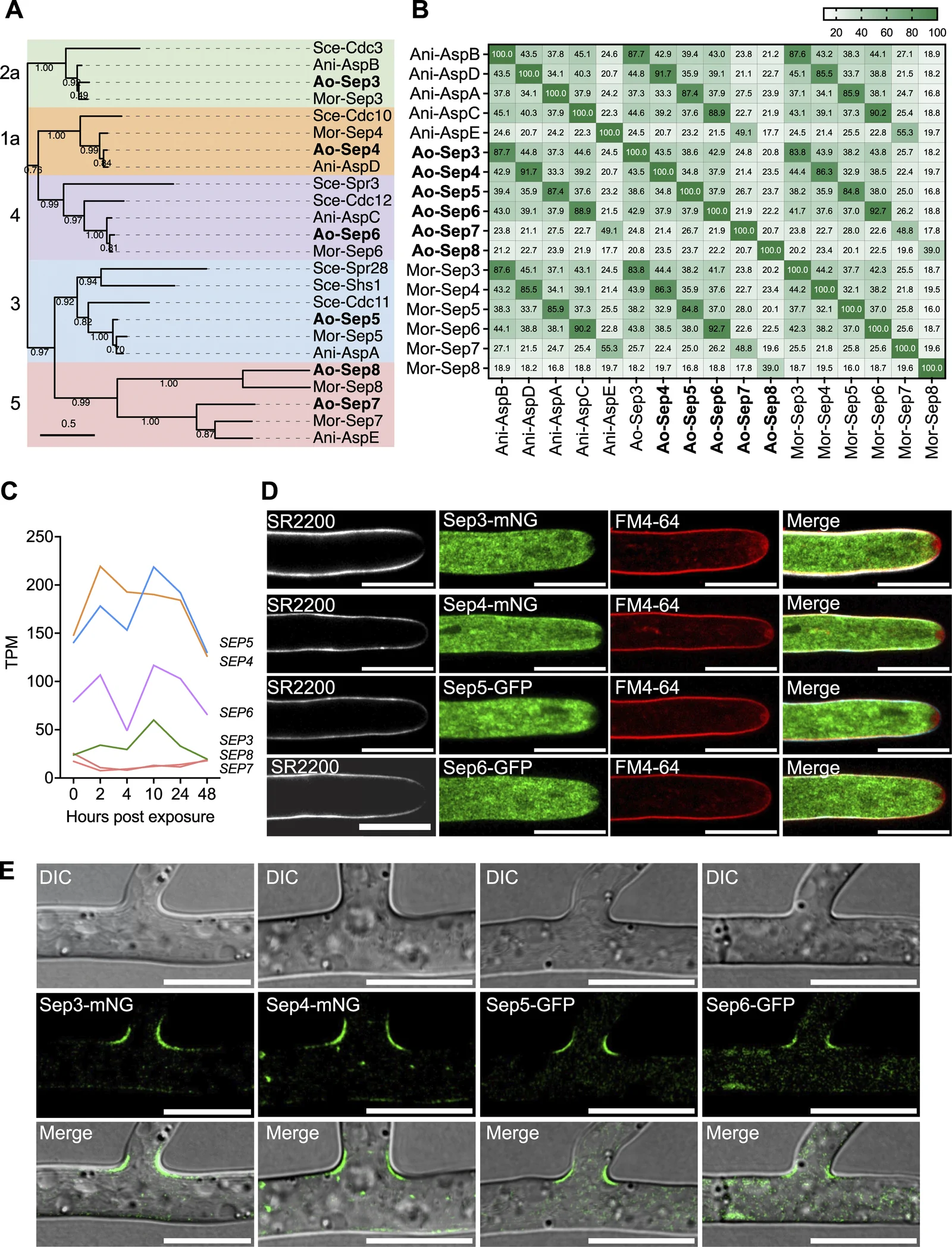
**Septins are evolutionarily conserved and localize to curved hyphal branch bases in *A. oligospora*. (A)** Maximum-likelihood phylogeny of fungal septins. Numbers on the left denote the septin classes. Node numbers indicate support values ranging from 0 to 1. The scale bar represents 0.5–amino acid substitutions per site. Species abbreviations are as defined in Fig. S1. **(B)** Pairwise amino acid sequence identity among fungal septins. Numbers indicate sequence identity percentages. **(C)** TPM values of differentially expressed septins in a published RNA-seq time course following exposure of *A. oligospora* to *C. elegans* (Lin et al., 2023). Values represent the mean of three biological replicates. **(D)** Localization of Sep3-mNG, Sep4-mNG, Sep5-GFP, and Sep6-GFP in vegetative hyphae. Septins exhibit diffuse localization at the hyphal apex. Hyphae were stained with SR2200 to visualize cell walls and FM4-64 to label the plasma membrane and the SPK. Merged images display SR2200, GFP or mNG, and FM4-64 fluorescence. **(E)** Localization of Sep3-mNG, Sep4-mNG, Sep5-GFP, and Sep6-GFP at hyphal branch bases. Merged images show mNG or GFP fluorescence overlaid with DIC images. All scale bars are 10 μm. Images are representative of three independent biological replicates. TPM, transcripts per kilobase million.

Given the role of septins in membrane remodeling and cell morphology (Benoit et al., 2023), we analyzed the *A. oligospora* genome and identified six septin genes (Fig. S3 A and Table S1). Sep3, Sep4, Sep5, and Sep6 belong to the four core septin groups (1a, 2a, 3, and 4), while Sep7 and Sep8 belong to the fifth group (5), which is unique to filamentous fungi and protists (Shuman and Momany, 2022). Sequence analyses showed that the core septins are highly conserved, sharing 85–90% sequence identity with orthologs in *A. nidulans* and *M. oryzae*, whereas Sep7 and Sep8 are much less conserved (Fig. S3 B). Analysis of a previously published time course RNA-seq dataset (Lin et al., 2023) revealed that the core septins are all upregulated in response to *C. elegans*, suggesting a role in trap morphogenesis and development (Fig. S3 C).

To examine their localization, we tagged the core septins with GFP or mNG and expressed them under endogenous promoters. In the fungal hyphal tips, we found that the core septins displayed strong diffuse signal in the cytosol but were excluded from the SPK, resulting in a distinct central void within the apical septin distribution (Fig. S3 D). They also localized at hyphal branching sites, accumulating at the base of nascent lateral hyphae where the curvature of membrane is pronounced (Fig. S3 E). Similar localization at curved membrane regions has been reported in the hyphae of *A. nidulans* and *Ashbya gossypii* (Bridges et al., 2016; Hernández-Rodríguez et al., 2014), suggesting that the recruitment of septin complexes to curved membrane domains is a conserved feature of filamentous fungal morphogenesis.

### Trap morphogenesis proceeds through sequential reorientation of the hyphal tip

**Figure 1.**
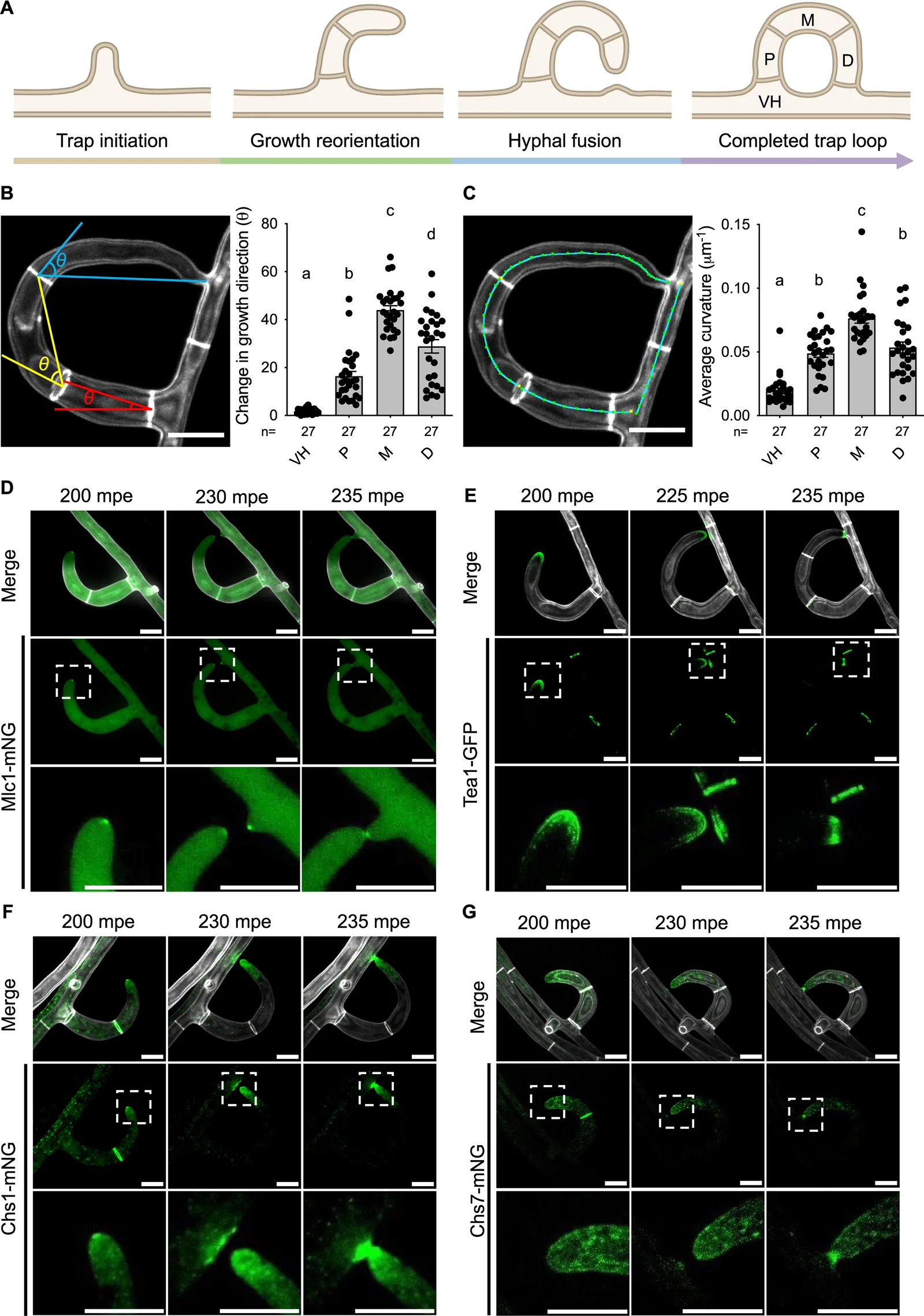
**Characterization of trap development reveals growth reorientation and dynamic redistribution of polarity proteins. (A)** Schematic representation of trap loop development, beginning with trap initiation, followed by growth reorientation, hyphal fusion, and completion of a three-celled trap loop comprising the proximal (P), middle (M), and distal (D) trap cells that remains attached to the vegetative hypha (VH). **(B)** Left, schematic illustrating quantification of growth direction changes during trap development. The angle (θ) between the initial and final growth directions was measured for each cell. Quantification of growth direction changes in VH and individual trap cells. **(C)** Left, representative image illustrating trap cell curvature analysis. Cell centerlines were traced and fitted with cubic B-splines (cyan), and average curvature was quantified. Right, quantification of average curvature in VH and individual trap cells. For B and C, bars represent the mean ± SEM; *n* values are indicated on the x axis and represent the number of cells analyzed. Groups labeled with different letters are significantly different, whereas groups sharing the same letter are not significantly different, based on one-way ANOVA followed by uncorrected Fisher’s LSD multiple-comparisons test (P < 0.05). **(D–G)** Localization of the cell polarity marker Mlc1-mNG (D), the cell-end marker Tea1-GFP (E), and the CHSs Chs1-mNG (F) and Chs7-mNG (G) during trap development. Merged images show GFP or mNG fluorescence overlaid with SR2200-stained cell walls. Enlarged regions are indicated by white dashed rectangles in the lower magnification images. Scale bars, 10 μm. Images in all panels are representative of three independent biological replicates.

Nematode-derived signals trigger the transition of *A. oligospora* from vegetative growth to a predatory lifestyle by inducing trap formation. Trap development begins with the emergence of a lateral branch from a vegetative hypha (trap initiation). As the branch elongates, its tip progressively reorients and bends toward the original hypha (growth reorientation). Upon contact, the two hyphae undergo fusion (hyphal fusion), completing a closed loop that differentiates into a three-celled trap consisting of proximal (P), middle (M), and distal (D) cells, while remaining attached to the vegetative hypha (VH) (Fig. 1 A). To quantitatively characterize trap morphogenesis, we measured changes in growth direction relative to the initial growth axis (Fig. 1 B). All three trap cells exhibited significantly larger directional changes than vegetative hyphae, with the middle trap cell showing the greatest deviation from its original growth trajectory (Fig. 1 B). We then quantified cell curvature by spline-fitted cell centerlines (Fig. 1 C). Consistent with the directional changes, trap cells were significantly more curved than vegetative hyphae, and the middle trap cell exhibited the highest curvature (Fig. 1 C). These analyses indicate that trap morphogenesis involves coordinated reorientation of hyphal growth and localized bending, with the middle trap cell undergoing the most pronounced morphological remodeling during trap loop formation.

### Polarity cues and cytoskeletal dynamics drive trap loop formation

To investigate how polarity is established during trap morphogenesis, we performed time-lapse live-cell imaging to monitor the dynamics of polarity markers, CHSs, and cytoskeletal components. During trap elongation, Mlc1-mNG localized to the growing trap apex, whereas Tea1-mNG was enriched at the apical cortex and at newly formed septa (Fig. 1, D and E; and Video 2). Chs1 and Chs7 exhibited localization patterns similar to that of Mlc1, while also accumulating at sites of septum formation (Fig. 1, F and G; and Video 2). As the growing trap hypha approached the original vegetative hypha, ∼230 min post-nematode exposure (mpe), all four proteins became concentrated at the prospective fusion site (Fig. 1, D–G; and Video 2). These observations indicate that polarized growth machinery and CHSs are coordinately recruited to the trap apex, septa, and the future fusion site throughout trap morphogenesis.

**Video 2.** **Cell polarity proteins are recruited to hyphal tip and fusion sites during trap formation.** Time-lapse fluorescence imaging of an *A. oligospora* expressing Tea1-GFP, Mlc1-mNG, Chs1-mNG, or Chs7-mNG and costained with SR2200 (white) during trap development following 3 h of nematode exposure. This video corresponds to the micrographs shown in Fig. 1, D–G. Images were acquired every 5 min and are displayed at 2 frames per second. The time stamp indicates the time elapsed since exposure to *C. elegans* and is displayed in HH:MM:SS format. Scale bar, 20 μm.

**Figure 2.**
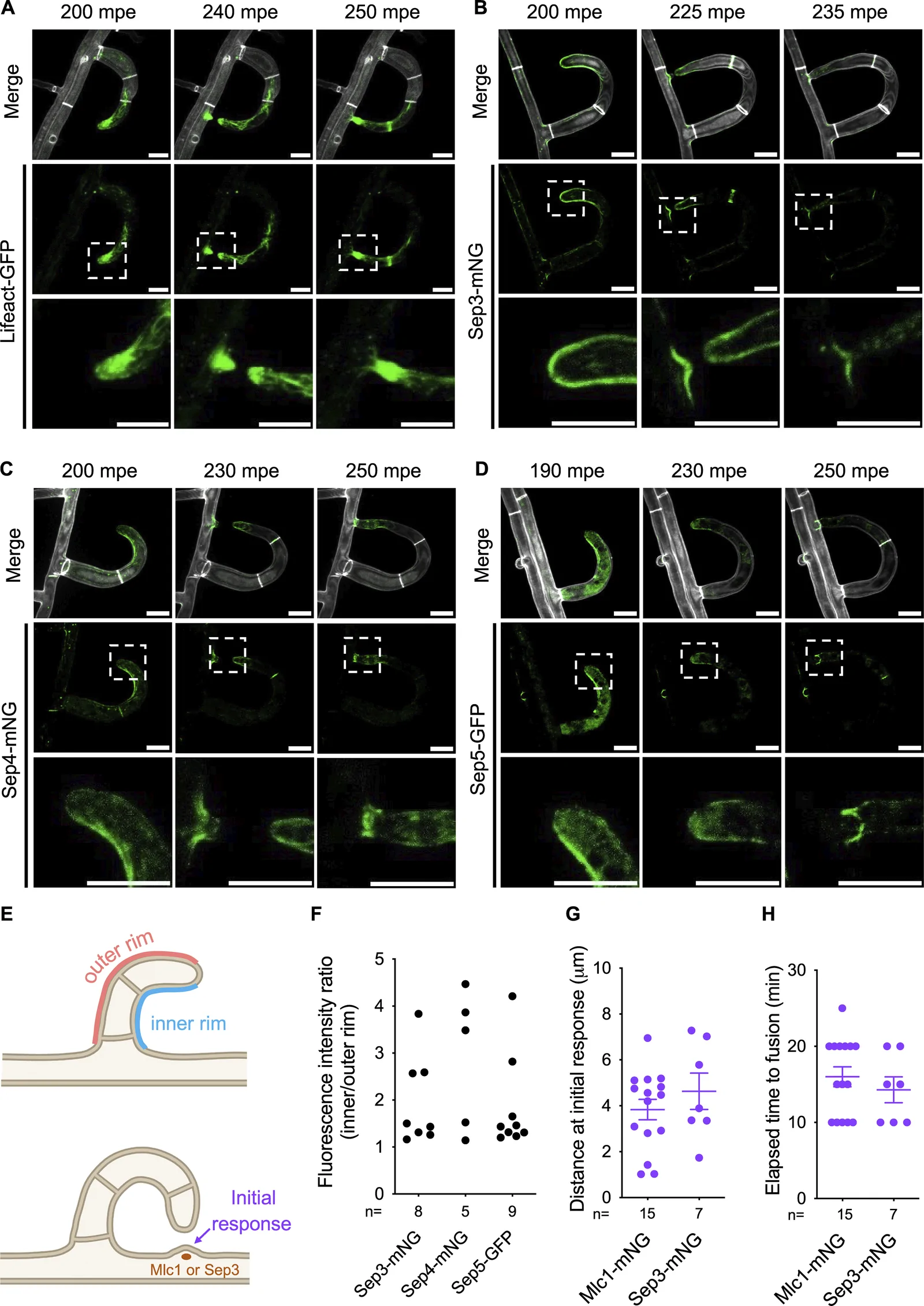
**Dynamic actin and asymmetric septin localization during trap morphogenesis and hyphal fusion. (A–D)** Localization of Lifeact-GFP (A), Sep3-mNG (B), Sep4-mNG (C), and Sep5-GFP (D) during trap formation following 3 h of nematode exposure. Merged images show GFP or mNG fluorescence overlaid with SR2200-stained cell walls. Enlarged regions are indicated by white dashed rectangles in the lower magnification images. Scale bars, 10 μm. Images in all panels are representative of three independent biological replicates. **(E)** Schematics illustrating the measurements used for quantitative analysis. Top, fluorescence intensity was measured along the entire inner (aqua) and outer rims (salmon) of the curved developing trap loop. Bottom, Mlc1-mNG and Sep3-mNG dynamics at the prospective fusion site were monitored to identify the onset of the receiving hyphal response to the approaching trap hypha. **(F)** Quantification of the fluorescence intensity ratio between the inner and outer trap rims for Sep3-mNG, Sep4-mNG, and Sep5-GFP. Individual data points represent individual traps. **(G)** Quantification of the distance between the approaching trap hypha and the receiving hypha when Mlc1-mNG or Sep3-mNG first appeared at the prospective fusion site. **(H)** Quantification of the elapsed time from the onset of the receiving hyphal response to hyphal fusion. For G and H, data represent the mean ± SEM; *n* values are indicated on the x axis and represent the number of fusion events analyzed. mpe, minutes post-nematode exposure.

We next examined the dynamics of the actin and septin cytoskeletons during trap formation. During trap elongation, Lifeact-GFP marked a prominent actin network at the growing apex (Fig. 2 A and Video 3). In contrast, septins displayed a striking asymmetric distribution, preferentially accumulating along the inner, concave side of the developing trap loop (Fig. 2, B–D and Video 4). To quantify this asymmetry, we measured the fluorescence intensity along the entire inner and outer rims of the curved trap loop (Fig. 2 E, top). Approximately 20–50% of traps exhibited an inner-to-outer fluorescence intensity ratio of >2, whereas the remaining traps showed relatively little difference between the two sides (Fig. 2 F). As hyphal fusion commenced, both actin and septins also accumulated at the prospective fusion site (Fig. 2, A–D; and Videos 3 and 4). These findings reveal that remodeling of the polarity machinery and asymmetric septin assembly coordinate to redirect hyphal growth toward loop closure during trap morphogenesis.

**Video 3.** **F-actin accumulates at hyphal tip and fusion site during trap formation.** Time-lapse fluorescence imaging of an *A. oligospora* expressing Lifeact-GFP and costained with SR2200 (white) during trap development following 3 h of nematode exposure. This video corresponds to the micrographs shown in Fig. 2 A. Images were acquired every 5 min and are displayed at 2 frames per second. The time stamp indicates the time elapsed since exposure to *C. elegans* and is displayed in HH:MM:SS format. Scale bar, 20 μm.

**Video 4.** **Septins form an asymmetric inner-rim scaffold during trap formation.** Time-lapse fluorescence imaging of an *A. oligospora* expressing Sep3-mNG, Sep4-mNG, Sep5-GFP, or Sep6-GFP and costained with SR2200 (white) during trap development following 3 h of nematode exposure. This video corresponds to the micrographs shown in Fig. 2, B–D. Images were acquired every 5 min and are displayed at 2 frames per second. The time stamp indicates the time elapsed since exposure to *C. elegans* and is displayed in HH:MM:SS format. Scale bar, 20 μm.

Hyphal fusion is initiated through cell–cell communication between the two fusion partners (Daskalov et al., 2017). To determine when the receiving hypha first responds to the approaching trap hypha, we monitored Mlc1 and Sep3 dynamics at the prospective fusion site during trap closure and observed that Mlc1-mNG and Sep3-mNG first accumulated at the prospective fusion site when the trap and receiving hyphae were separated by ∼4 μm (Fig. 2, E and G). Hyphal fusion then was completed ∼15 min later (Fig. 2 H). These observations suggest that mature trap loop formation requires short-range cell–cell communication between the trap apex and receiving hypha to coordinate cell fusion.

### Infection bulbs transition from isotropic growth to multipolar growth to generate invasive hyphae

**Figure 3.**
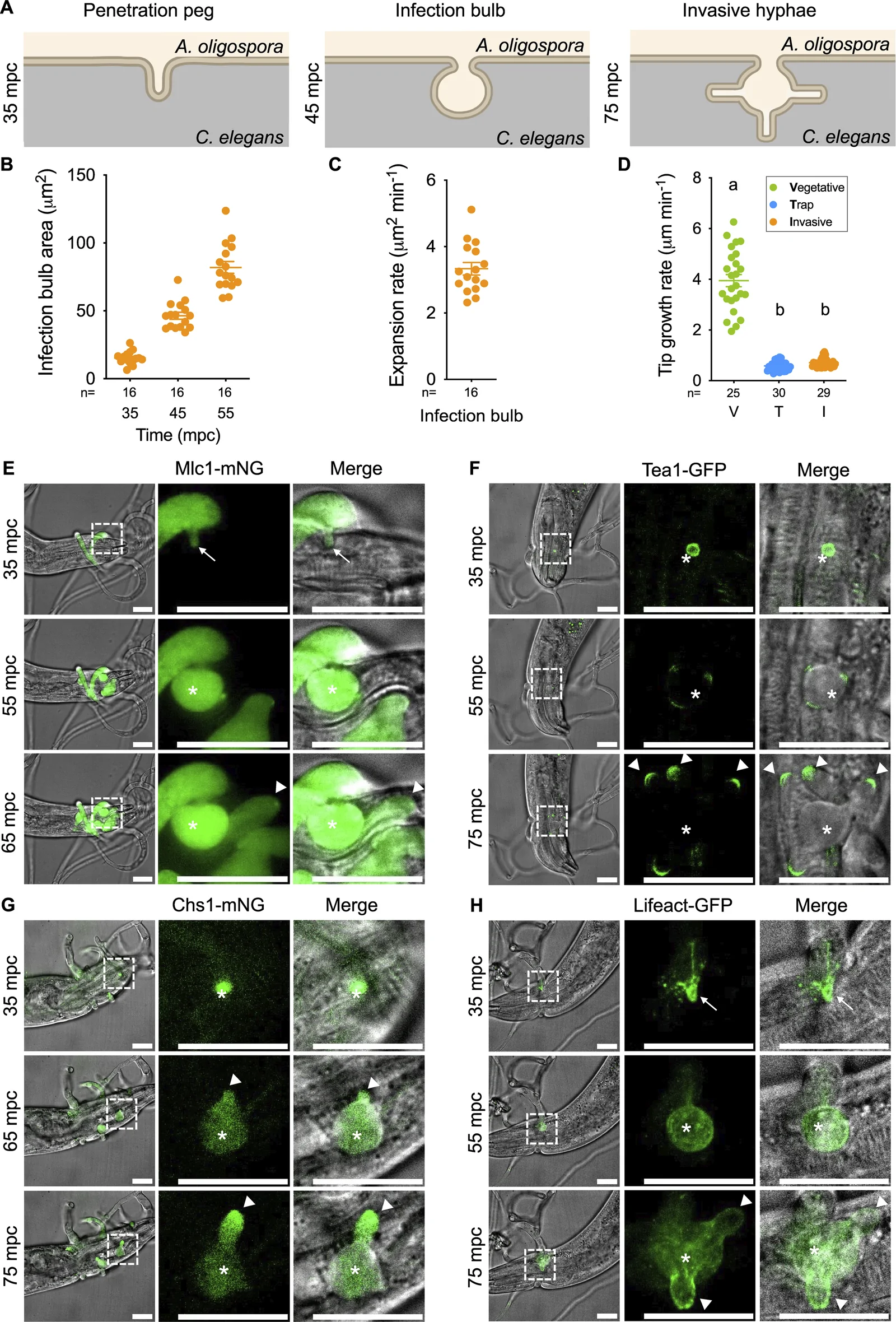
**Characterization of infection structure development reveals the transition from isotropic infection bulb expansion to multipolar invasive hyphal growth. (A)** Schematic representation of three sequential stages of infection structure development from 35 to 75 mpc. Infection structure development begins with penetration peg formation, followed by infection bulb formation and invasive hyphal growth. **(B–D)** Quantification of infection bulb area (B), infection bulb expansion rate (C), and hyphal tip growth rate in different cell types (D). Data represent the mean ± SEM; *n* values are indicated on the x axis. For B and C, *n* represents the number of infection bulbs analyzed; for D, *n* represents the number of hyphal tips analyzed. Groups labeled with different letters are significantly different, whereas groups sharing the same letter are not significantly different, based on one-way ANOVA followed by uncorrected Fisher’s LSD multiple-comparisons test (P < 0.05). **(E–H)** Localization of Mlc1-mNG (E), Tea1-GFP (F), Chs1-mNG (G), and Lifeact-GFP (H) during infection bulb and invasive hyphal development. Time-lapse imaging was performed from 35 to 75 mpc. Penetration pegs (arrows), infection bulb (asterisks), and invasive hyphae (triangles) are indicated. Merged images show the mNG or GFP fluorescence overlaid with DIC images of the trapped nematodes. Enlarged regions are indicated by white dashed rectangles in the lower magnification images. Scale bars, 20 μm. Images are representative of three independent biological replicates. mpc, minutes post-nematode capture.

Following nematode capture, trap cells differentiate penetration pegs that breach the nematode cuticle and expand into infection bulbs. As infection bulbs mature, they undergo a transition to multipolar growth, giving rise to multiple invasive hyphae that colonize the nematode (Fig. 3 A). To quantitatively characterize infection structure development, we measured the rate of growth expansion of infection bulb and the rate of elongation of invasive hyphae. We observed that infection bulbs expanded rapidly, reaching an average area of ∼82 μm^2^ by 55 minutes post-nematode capture (mpc) with an average expansion rate of ∼3.3 μm^2^ min^−1^ (Fig. 3, B and C). Invasive hyphae elongated at ∼0.7 μm min^−1^, a rate comparable to that of trap hyphae (0.6 μm min^−1^) but substantially slower than vegetative hyphae (3.8 μm min^−1^) (Fig. 3 D).

To determine how polarity is established during infection structure development, we tracked the dynamics of polarity markers, CHSs, and the cytoskeleton by time-lapse live-cell imaging. During infection bulb expansion, Mlc1-mNG and Chs1-mNG were broadly distributed throughout the bulb, consistent with isotropic growth, whereas Tea1-GFP was enriched around the bulb periphery (Fig. 3, E–G and Video 5). As the infection bulb approached its maximum size, Tea1-GFP became concentrated at discrete cortical sites that preceded invasive hyphal emergence (Fig. 3 F and Video 5). Following branch initiation, Mlc1, Chs1, and Tea1 all accumulated at invasive hyphal tips (Fig. 3, E–G; and Video 5). These observations indicate that infection bulb development involves a transition from diffuse polarity during isotropic expansion to the establishment of multiple discrete polarity sites that direct invasive hyphal outgrowth.

**Video 5.** **Spatiotemporal reorganization of polarity markers during the transition from the infection bulb to invasive hyphae.** Time-lapse fluorescence video of *A. oligospora* expressing Mlc1-mNG, Tea1-GFP, or Chs1-mNG (green) and costained with SR2200 (white) during infection bulb and invasive hyphal development. This video corresponds to the micrographs shown in Fig. 3, E–G. Images were acquired every 5 min and are displayed at 2 frames per second. The time stamp indicates the time elapsed since *C. elegans* capture and is displayed in HH:MM:SS format. Scale bar, 20 μm.

**Figure 4.**
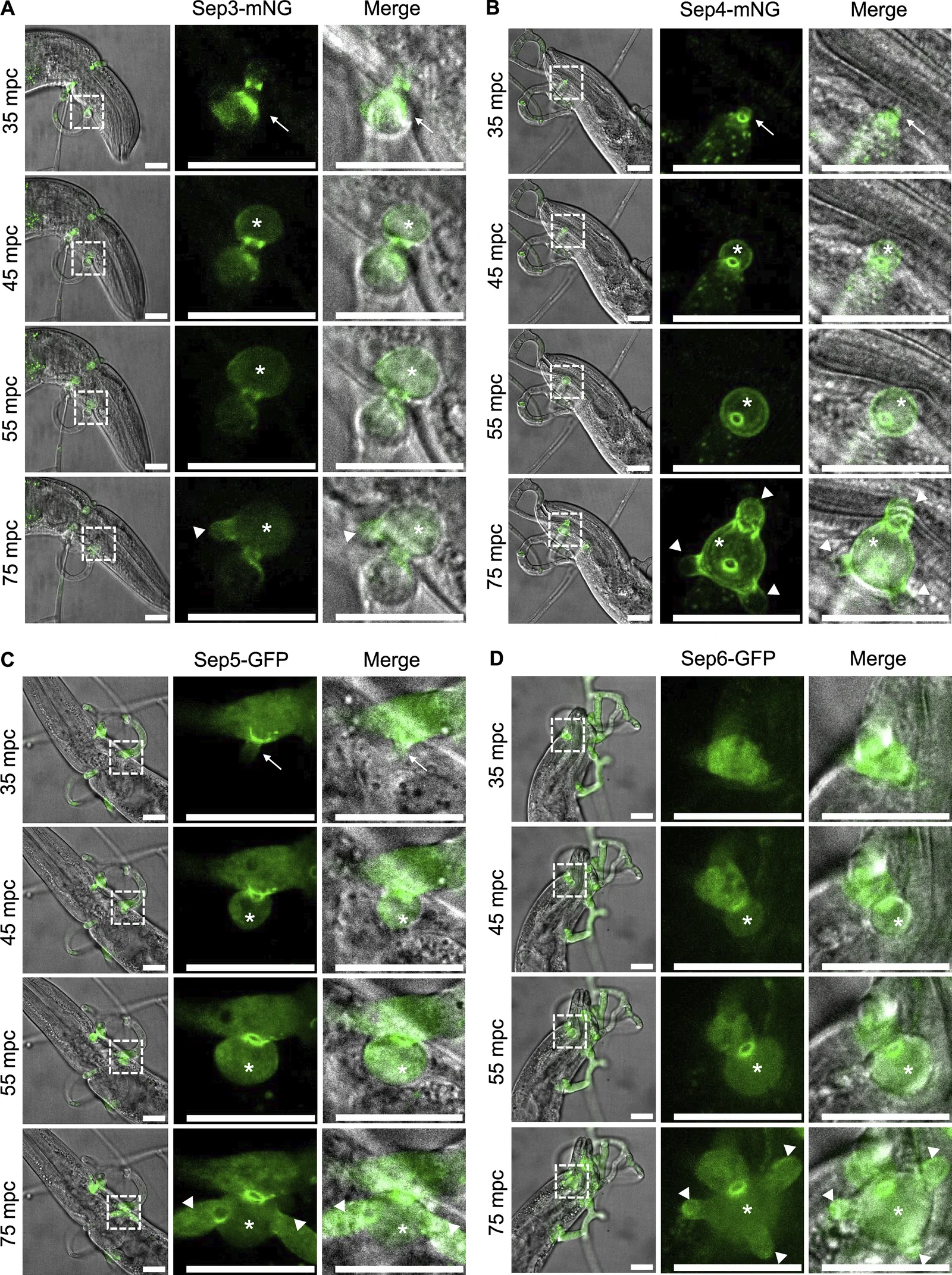
**Septins assemble into a ring structure at the base of the infection bulb. (A–D)** Localization of Sep3-mNG (A), Sep4-mNG (B), Sep5-GFP (C), and Sep6-GFP (D) during infection bulb and invasive hypha development. Time-lapse imaging was performed from 35 to 75 mpc. Penetration pegs (arrows), infection bulb (asterisks), and invasive hyphae (triangles) are indicated. Merged images show the mNG or GFP fluorescence overlaid with DIC images of the trapped nematodes. Enlarged regions are indicated by white dashed rectangles in the lower magnification images. Scale bars, 20 μm. Images are representative of three independent biological replicates. mpc, minutes post-nematode capture.

We next examined how the actin and septin cytoskeletons are reorganized during infection structure development. Both cytoskeletal systems initially assembled into ringlike structures at sites that subsequently developed into infection bulbs (Fig. 3 H, Fig. 4; and Videos 6 and 7). As the infection bulb expanded, actin and septin signals became broadly distributed throughout the bulb, while a cortical ring persisted at its base (Fig. 3 H, Fig. 4; and Videos 6 and 7). During invasive hyphal emergence, actin became concentrated at the tips of nascent invasive hyphae (Video 6), whereas septins relocalized to the junction between the infection bulb and invasive hyphae (Fig. 4 and Video 7). Thus, the transition from isotropic bulb expansion to multipolar invasive growth is accompanied by spatial segregation of the actin and septins.

**Video 6.** **F-actin dynamics during infection structure maturation and invasive growth.** Time-lapse fluorescence video of *A. oligospora* expressing Lifeact-GFP (green) and costained with SR2200 (white) during infection bulb and invasive hyphal development. This video corresponds to the micrographs shown in Fig. 3 H. Images were acquired every 5 min and are displayed at 2 frames per second. The time stamp indicates the time elapsed since *C. elegans* capture and is displayed in HH:MM:SS format. Scale bar, 20 μm.

**Video 7.** **Septin dynamics during infection structure maturation and invasive growth.** Time-lapse fluorescence video of *A. oligospora* expressing Sep3-mNG, Sep4-mNG, Sep5-GFP, or Sep6-GFP (green) and costained with SR2200 (white) during infection bulb and invasive hyphal development. This video corresponds to the micrographs shown in Fig. 4. Images were acquired every 5 min and are displayed at 2 frames per second. The time stamp indicates the time elapsed since *C. elegans* capture and is displayed in HH:MM:SS format. Scale bar, 20 μm.

### CHS and cell-end markers coordinate trap morphogenesis

**Figure S4.**
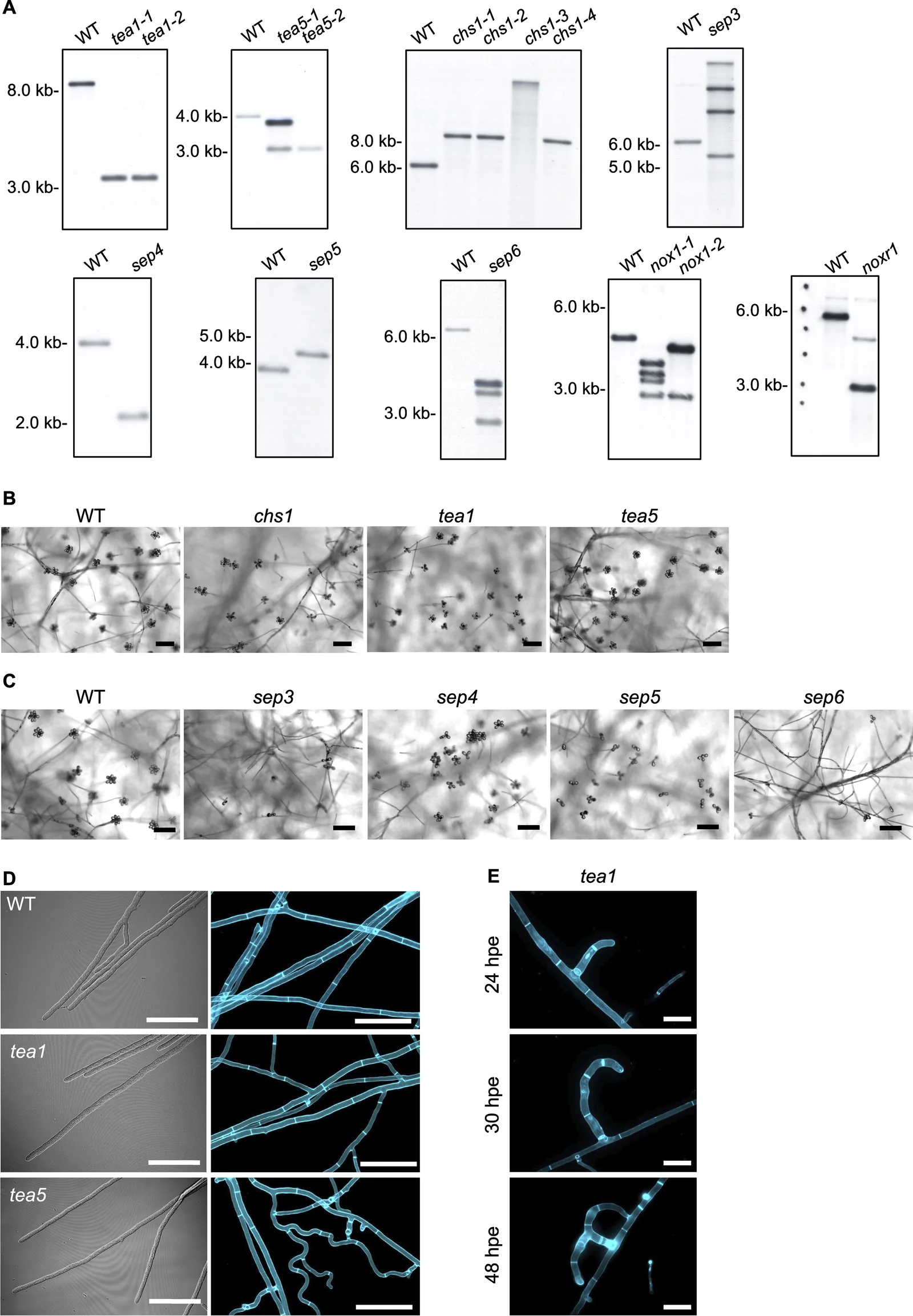
**Validation of gene deletion mutants and phenotypic analysis of *tea* mutants. (A)** Southern blot analysis confirming targeted deletion of *TEA1*, *TEA5*, *CHS1*, *SEP3*, *SEP4*, *SEP5*, *SEP6*, *NOX1*, and *NOXR1*. Genomic DNA from the WT and various mutant strains was digested with specific restriction enzymes and hybridized with probes targeted to examine events of ectopic integrations. **(B and C)** Representative conidiation phenotypes of the WT and the indicated mutants after 4 days of growth on PDA. Scale bar, 100 μm. **(D)** Left, representative DIC images of hyphal tips. Right, SR2200-stained hyphae of the WT, *tea1*, and *tea5* mutants. Scale bar, 50 μm. **(E)** Trap morphogenesis in the *tea1* mutant after 24, 30, and 48 h of continuous induction with 400 *C. elegans* L4 larvae. Traps were stained with SR2200. Scale bar, 20 μm. Images are representative of three independent biological replicates. hpe, hours post-nematode exposure.

**Figure 5.**
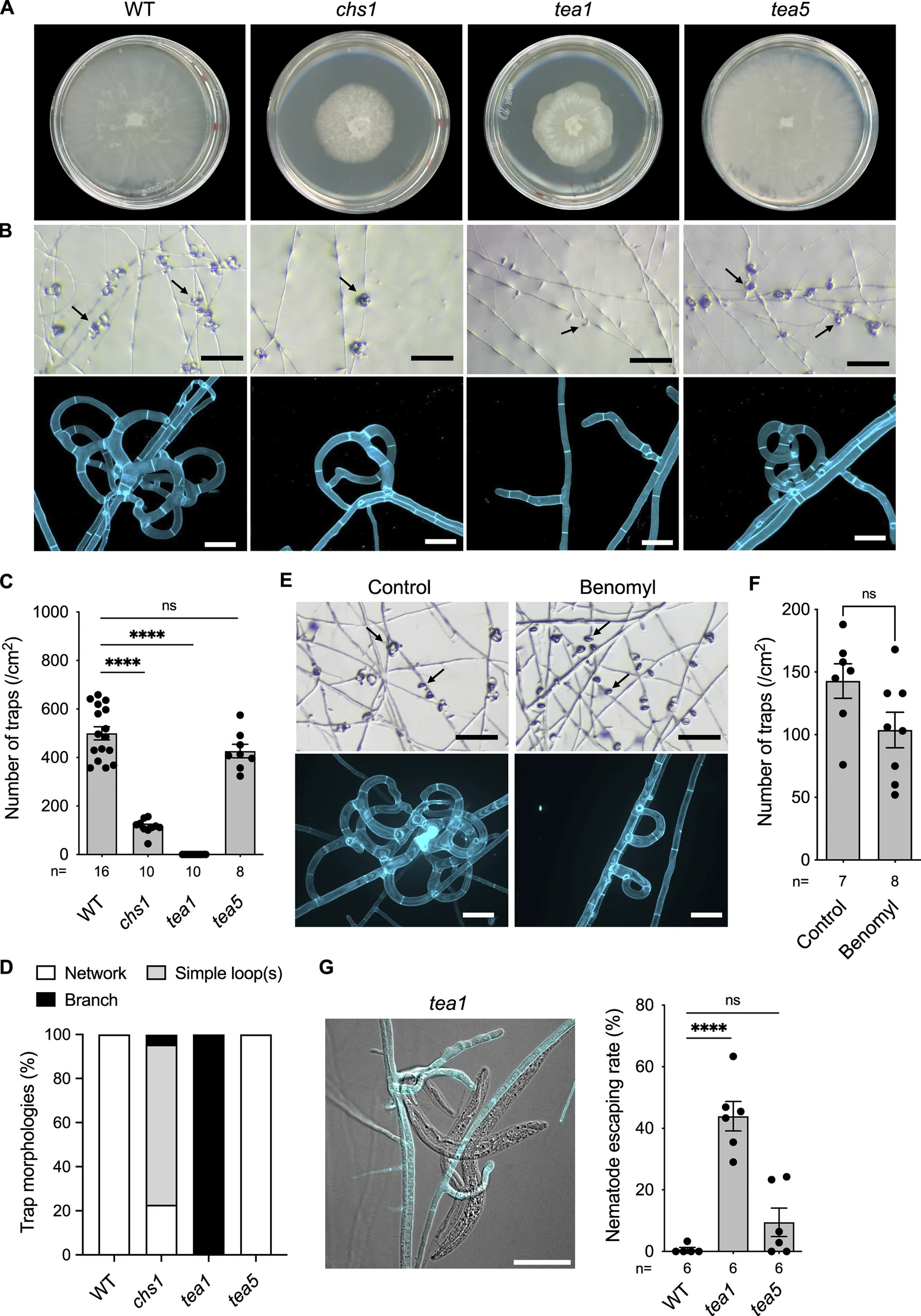
**CHS Chs1 and cell-end markers Tea1 and Tea5 play distinct roles in hyphal growth and trap morphogenesis in *A. oligospora*. (A)** Colonies of the WT, *chs1*, *tea1*, and *tea5* mutants grown on 5.5-cm PDA plates for 3 days. **(B)** Upper, representative bright-field images of the traps induced by *C. elegans* in the WT, *chs1*, *tea1*, and *tea5* mutants. The black arrows indicate traps. Scale bar, 200 μm. Lower, confocal images of SR2200-stained traps formed by the WT, *chs1*, *tea1*, and *tea5* strains after 24 h of continuous induction with 400 *C. elegans* larvae. Scale bar, 20 μm. **(C)** Quantification of trap numbers induced by *C. elegans* in the WT, *chs1*, *tea1*, and *tea5* mutants. **(D)** Distribution of trap morphologies in the WT, *chs1*, *tea1*, and *tea5* mutants. **(E)** Effect of benomyl on trap development in the WT. Upper, representative bright-field images of traps formed on LNM with or without benomyl following induction with 30 *C. elegans* L4 larvae. Black arrows indicate traps. Scale bar, 200 μm. Lower, confocal images of SR2200-stained traps following 24 h of continuous induction with 400 *C. elegans* larvae. Scale bar, 20 μm. **(F)** Quantification of trap numbers formed in the WT with or without benomyl following induction with 30 *C. elegans* L4 larvae. **(G)** Representative images of a nematode captured by an SR2200-stained *tea1* trap and quantification of nematode escaping rates in the WT, *tea1*, and *tea5* mutants. Scale bar, 50 μm. For C, F, and G, data represent the mean ± SEM; *n* values are indicated on the x axis and represent the number of fungal plates analyzed. Statistical significance was determined using a two-tailed unpaired Student’s *t* test. ****P < 0.0001; ns, not significant. Images are representative of three independent biological replicates.

To define the functional contributions of Chs1 and the cell-end markers Tea1 and Tea5, we generated target deletions and confirmed the genotypes via PCR and Southern blotting (Fig. S4 A). Both *chs1* and *tea1* mutants exhibited reduced vegetative growth on nutrient-rich medium (potato dextrose agar [PDA]), whereas conidiation was comparable to that of the wild type (WT) (Fig. 5 A and Fig. S4 B). Unlike the zigzag hyphae characteristic of *tea* mutants in *A. nidulans* (Takeshita et al., 2008), hyphal tips of the *tea1* and *tea5* mutants remained straight in *A. oligospora* (Fig. S4 D). Instead, the *tea5* mutant developed zigzag hyphae specifically within the colony interior, suggesting that Tea5 primarily contributes to hyphal network organization rather than apical growth in *A. oligospora*.

To determine how these proteins contribute to trap morphogenesis, we quantified trap formation following exposure to 30 *C. elegans* L4 larvae. The *chs1* mutant produced ∼100 traps cm^−2^, representing a fivefold reduction compared with the ∼500 traps cm^−2^ formed by the WT (Fig. 5, B and C). Moreover, *chs1* traps consisted predominantly of simple loops, indicating a defect in the maturation of complex trap networks (Fig. 5, B and D). The *tea1* mutant displayed a more severe morphogenetic phenotype: trap hyphae failed to reorient their growth toward the receiving hypha and instead continued to extend as uncurved branches, preventing timely loop closure (Fig. 5, B–D). Notably, prolonged incubation (48 h) eventually allowed some *tea1* trap hyphae to complete loop formation (Fig. S4 E), indicating that Tea1 is required for the efficiency and directionality, rather than the absolute capacity, of trap morphogenesis. In contrast, the *tea5* mutant formed trap numbers comparable to those of the WT but produced significantly smaller trap networks (Fig. 5, B–D).

In *S. pombe* and *A. nidulans*, Tea1 and its homolog TeaA are delivered to sites of polarized growth by microtubules (Behrens and Nurse, 2002; Takeshita et al., 2008). We therefore asked whether microtubules similarly contribute to trap morphogenesis in *A. oligospora*. Treatment with the microtubule polymerization inhibitor benomyl significantly reduced trap formation and impaired the development of complex trap networks (Fig. 5, E and F). However, unlike the *tea1* mutant, benomyl-treated hyphae remained capable of forming simple trap loops, indicating that disruption of microtubules only partially phenocopies the loss of Tea1. Consistent with its pronounced morphogenetic defects, the *tea1* mutant exhibited a significantly higher nematode escaping rate than the WT, whereas the *tea5* mutant retained trapping efficiency comparable to that of the WT (Fig. 5 G).

Collectively, these findings demonstrate Tea1 directs the hyphal reorientation required for trap loop formation, while Chs1 promotes trap maturation, and Tea5 contributes primarily to vegetative hyphal organization.

### Septins differentially regulate vegetative growth and trap morphogenesis

**Figure 6.**
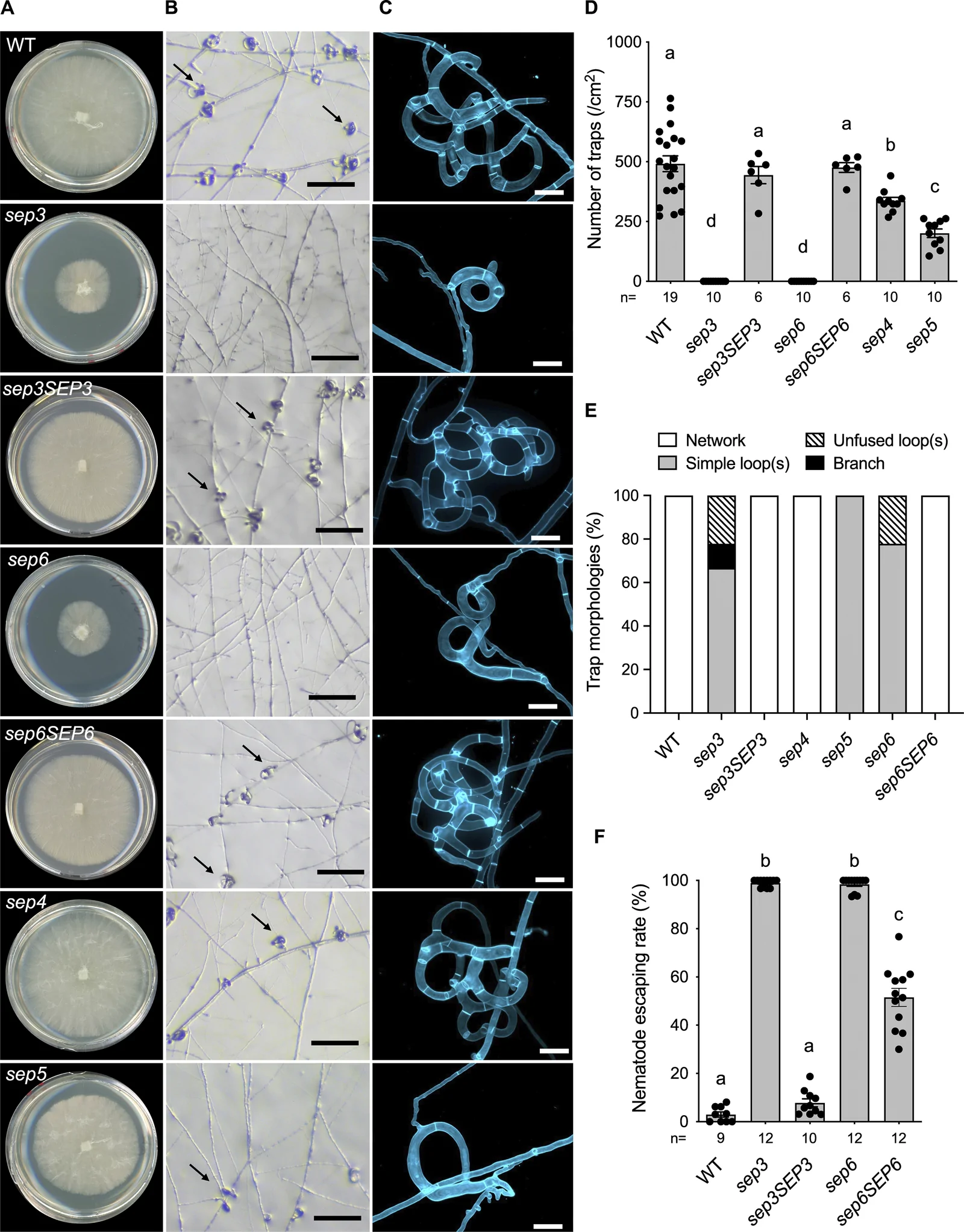
**Core septins differentially regulate vegetative growth, conidiation, and trap morphogenesis in *A. oligospora*. (A)** Colony morphology of the WT, *sep* mutants, and the *sep3* and *sep6* complementation lines grown on 5.5-cm PDA plates for 3 days. **(B)** Representative bright-field images of traps induced by *C. elegans* in the WT, *sep* mutants, and the *sep3* and *sep6* complementation lines. Black arrows indicate traps. Scale bars, 200 μm. **(C)** Confocal images of SR2200-stained traps formed by the WT, *sep* mutants, and the *sep3* and *sep6* complementation lines after 24 h of continuous induction with 400 *C. elegans* L4 larvae. Scale bars, 20 μm. Images are representative of three independent biological replicates. **(D)** Quantification of trap numbers induced by 30 *C. elegans* L4 larvae in the WT, *sep* mutants, and the *sep3* and *sep6* complementation lines. **(E)** Distribution of trap morphologies in the WT, *sep* mutants, and the *sep3* and *sep6* complementation lines. **(F)** Quantification of nematode escaping rates in the WT, *sep* mutants, and the *sep3* and *sep6* complementation lines. For D and F, data represent the mean ± SEM; *n* values are indicated on the x axis and represent the number of fungal plates analyzed. Groups labeled with different letters are significantly different, whereas groups sharing the same letter are not significantly different, based on one-way ANOVA followed by uncorrected Fisher’s LSD multiple test (P < 0.05).

To investigate the functions of septins in *A. oligospora*, we generated deletion mutants of the four core septins and confirmed the mutant genotypes by PCR and Southern blotting (Fig. S4 A). The *sep3* and *sep6* mutants exhibited severe defects in vegetative hyphal growth and conidiation, whereas the *sep5* mutant exhibited only mild growth defects, and the *sep4* strain was indistinguishable to the WT (Fig. 6 A and Fig. S4 C). Complementation of *SEP3* and *SEP6* restored WT growth and conidiation, confirming that these defects resulted from loss of the respective septin genes (Fig. 6 A).

Given the asymmetric localization of septins along the inner rim of developing traps, we next investigated how they contribute to trap morphogenesis. Deletion of any core septin significantly reduced trap formation in response to *C. elegans* (Fig. 6, B–E). The *sep3* and *sep6* mutants barely formed traps, while trap number was reduced but not eliminated in *sep4* and *sep5* (Fig. 6 D). Traps produced by the *sep3*, *sep5*, and *sep6* mutants consisted predominantly of simple or incomplete loops, whereas the traps of *sep4* mutant resembled the morphology of WT (Fig. 6, B–E). Complementation of *SEP3* and *SEP6* restored both trap formation and morphology (Fig. 6, B–E). Consistent with these developmental defects, *sep3* and *sep6* mutants exhibited significantly higher nematode escaping rates than the WT, and complementation restored trapping efficiency (Fig. 6 F). Collectively, these findings show that septins contribute to trap formation, with Sep3, Sep5, and Sep6 playing predominant roles in regulating network morphogenesis of the traps.

### Nox1 signaling regulates hyphal fusion during trap loop closure by recruiting polarity factors to the prospective fusion site

**Figure S5.**
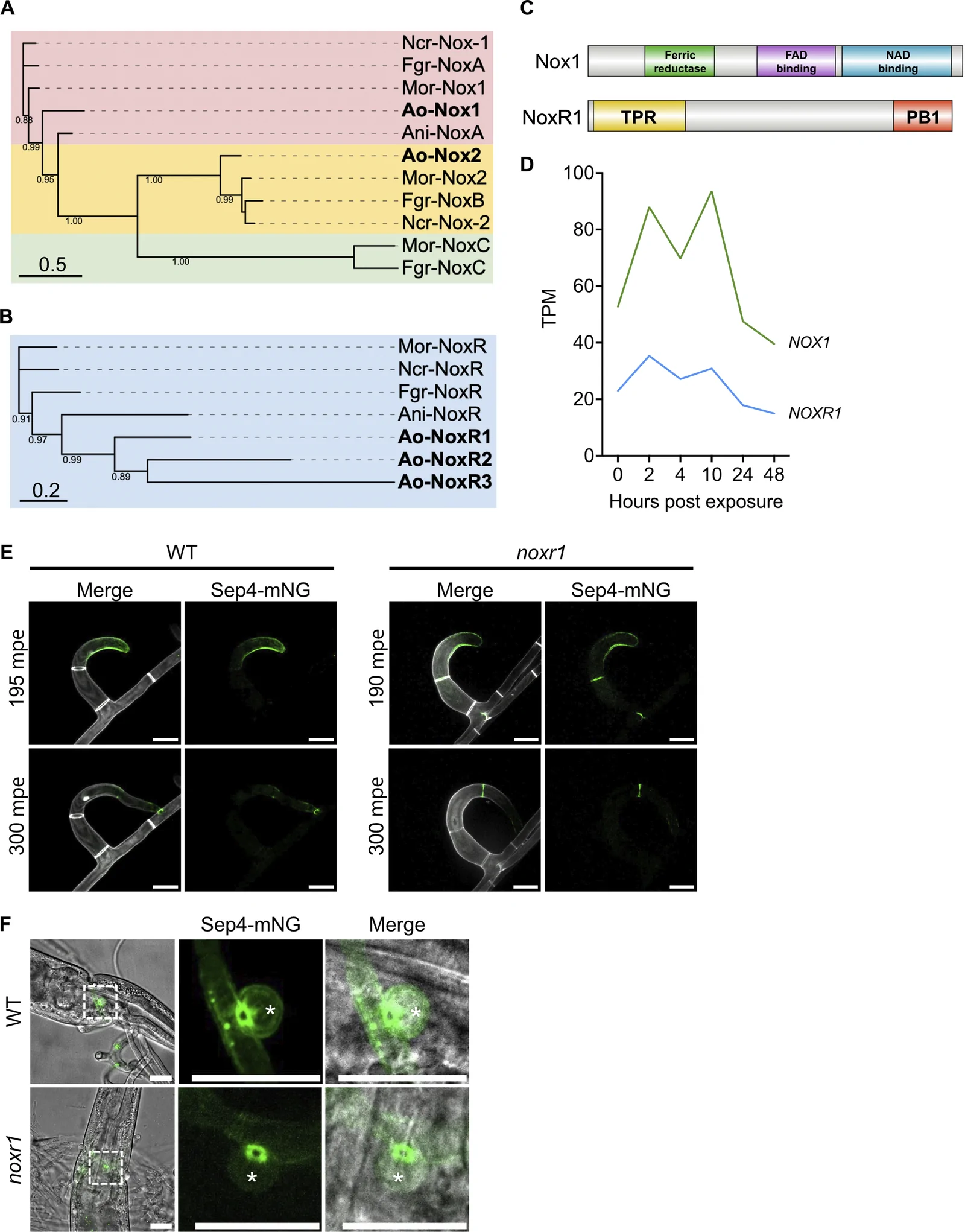
**Nox1 and NoxR1 are conserved and induced during trap morphogenesis but are dispensable for septin assembly in *A. oligospora*. (A and B)** Maximum-likelihood phylogenies of fungal Nox enzymes (A) and NoxR1 proteins (B). Node numbers indicate local support values ranging from 0 to 1. The scale bars represent 0.5– and 0.2–amino acid substitutions per site, respectively. Species abbreviations: Ani, *A. nidulans*; Ao, *A. oligospora*; Fgr, *F. graminearum*; Mor, *M. oryzae*; Ncr, *N. crassa*. **(C)** Domain architecture of Nox1 and NoxR1 from *A. oligospora*. Nox1 contains a ferric reductase transmembrane component–like domain (IPR013130), a FAD-binding 8 domain (IPR013112), and a ferric reductase NAD-binding domain (IPR013121). NoxR1 contains TPR (IPR011990) and a Phox and Bem1 (PB1; IPR000270) domain. **(D)** TPM values of differentially expressed *NOX1* and *NOXR1* in a published RNA-seq time course following exposure of *A. oligospora* to *C. elegans* (Lin et al., 2023). Values represent the average of three independent biological replicates. **(E)** Localization of Sep4-mNG in the WT and *noxr1* mutant during trap development from 190 to 300 min after *C. elegans* exposure. Merged images display mNG fluorescence overlaid with SR2200-stained cell walls. Scale bar, 10 μm. **(F)** Localization of Sep4-mNG in the WT and *noxr1* mutant during infection bulb development at 55 mpc. Asterisks indicate infection bulbs. Merged images show mNG fluorescence overlaid with DIC image of the trapped nematodes. Enlarged regions are indicated by white dashed rectangles in the lower magnification images. Scale bars, 20 μm. Images represent three independent biological replicates. mpe, minutes post-nematode exposure TPM, transcripts per kilobase million; TPR, tetratricopeptide repeat.

Given the conserved Nox1 signaling regulates cell fusion in fungi (Dirschnabel et al., 2014; Nordzieke et al., 2019; Roca et al., 2012), we next investigated whether Nox1 signaling regulates hyphal fusion during trap loop closure. The genome of *A. oligospora* encodes conserved homologs of Nox1, Nox2, but lacks a Nox3 homolog, and three regulatory subunits NoxR1, NoxR2, and NoxR3 (Fig. S5, A and B). Domain analysis showed that Ao-Nox1 and NoxR1 both contain the typical domains that mediate Nox complex assembly and activation in fungi and animals (Fig. S5 C) (Koga et al., 1999; Takemoto et al., 2011).

**Figure 7.**
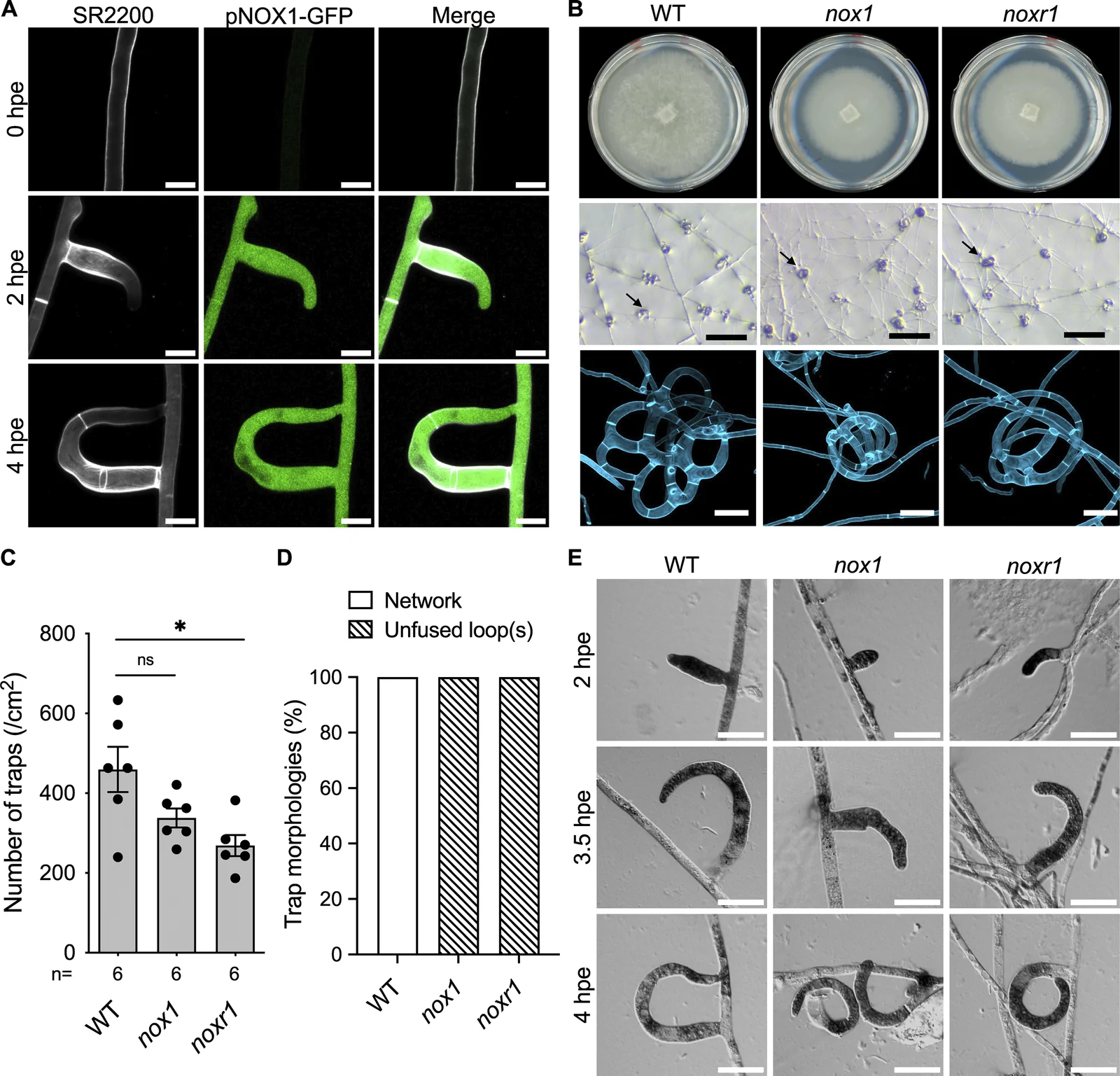
**Nox1 and NoxR1 are required for cell fusion during trap morphogenesis in *A. oligospora*. (A)** Localization of pNOX1-GFP at 0, 2, and 4 h after *C. elegans* exposure. Merged images show GFP fluorescence overlaid with SR2200-stained cell walls. Scale bars, 10 μm. **(B)** Upper, colonies of the WT and *nox* mutants grown on 5.5-cm PDA plates for 3 days. Middle, representative bright-field images of traps induced by *C. elegans* in the WT and *nox* mutants. Black arrows indicate traps. Scale bars, 200 μm. Lower, confocal images of SR2200-stained traps formed by the WT and *nox* mutants after 24 h of continuous induction with 400 *C. elegans* larvae. Scale bar, 20 μm. **(C)** Quantification of trap numbers induced by *C. elegans* in the WT and *nox* mutants. Data represent the mean ± SEM; *n* values are indicated on the x axis and represent the number of fungal plates analyzed. Statistical significance was determined using a two-tailed unpaired Student’s *t* test; *P < 0.05; ns, not significant. **(D)** Distribution of trap morphologies in the WT and *nox* mutants. **(E)** ROS production visualized by NBT staining in the WT and *nox* mutants at 2, 3.5, and 4 h after *C. elegans* exposure. Scale bar, 20 μm. Images are representative of three independent biological replicates. hpe, hours post-nematode exposure.

Transcriptional profiling following nematode exposure revealed that both *NOX1* and *NOXR1* were strongly upregulated during trap development (Fig. S5 D). We generated a GFP reporter driven by the endogenous *NOX1* promoter and observed strong expression of GFP after nematode exposure during trap formation (Fig. 7 A). These results indicate that the Nox1 complex is induced by nematode signals and coincides with trap morphogenesis.

To characterize the role of the Nox1 signaling during trap morphogenesis, we generated *nox1* and *noxr1* deletion mutants. Deletion of *NOX1* and *NOXR1* caused mild vegetative growth defects (Fig. 7 B). After exposure to nematodes, the *nox1* and *noxr1* mutants were able to develop similar number of traps compared with WT (Fig. 7 C). However, we observed that in these mutants, when the tip of the trap hyphal loop approached to the receiving hyphae, it failed to fuse upon contact (Fig. 7 B). Instead, the trap hyphal tip continued to elongate and curve, resulting in unfused, pigtail-shaped traps (Fig. 7 B). This phenotype was confirmed by quantitative analysis, which showed that nearly all mutant traps remained as unfused loops (Fig. 7 D).

To determine whether hyphal fusion defect in the *nox* mutants results from impaired ROS production, we monitored ROS accumulation using nitroblue tetrazolium (NBT) staining. In both WT and *nox* mutants, ROS accumulated at the growing trap tips during the early stages of trap development (2–3.5 hpe), indicating that ROS production was largely preserved (Fig. 7 E). However, when we examined the ROS signals at the prospective fusion site during hyphal fusion (4 hpe), we found that a discrete NBT signal accumulated at the prospective fusion site on the receiving hypha in WT, whereas this localized signal was absent in both *nox1* and *noxr1* mutants (Fig. 7 E). These findings indicate that Nox1 is required for the localized ROS production in the receiving hyphae at the prospective fusion site during trap loop closure.

**Figure 8.**
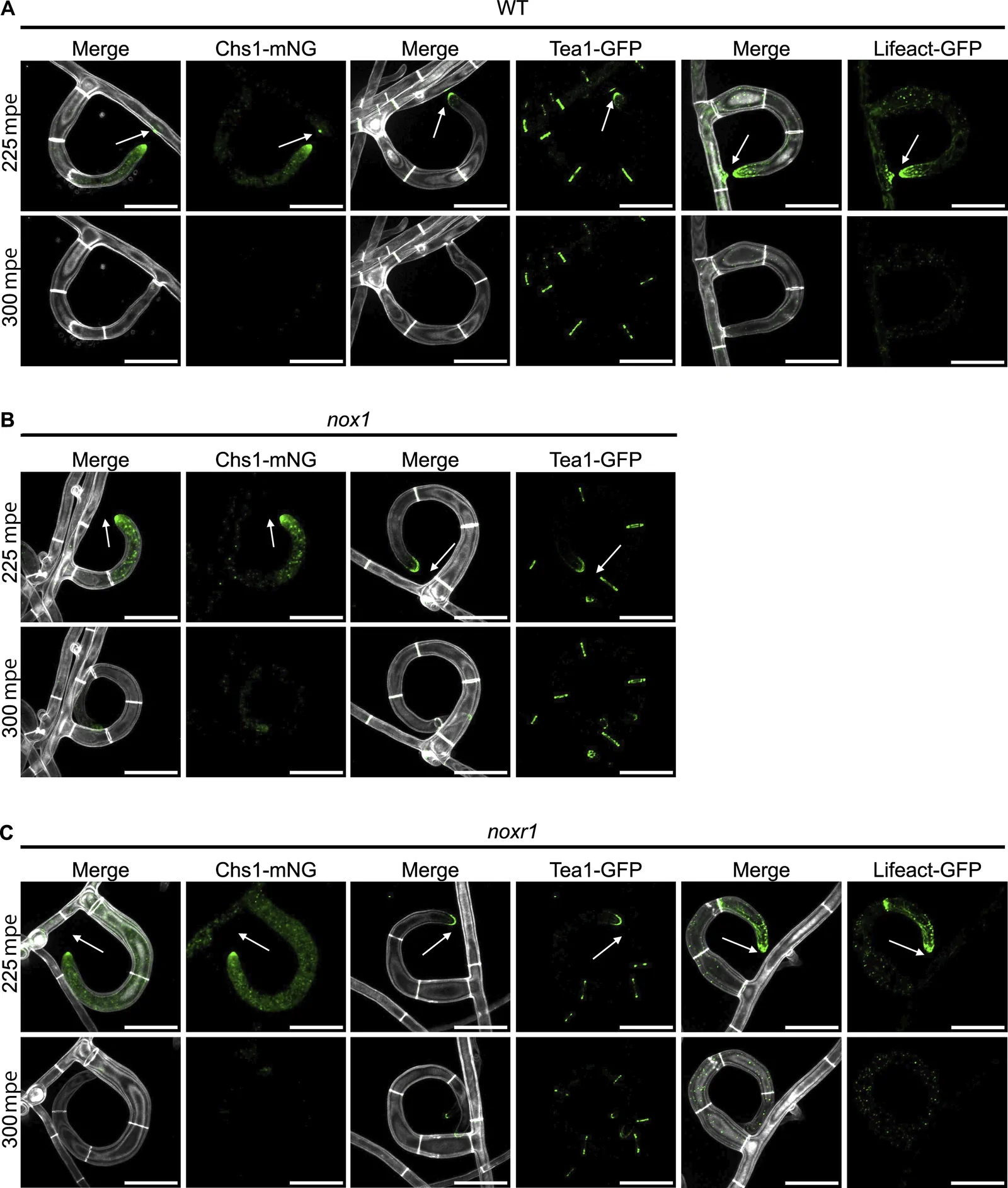
**Nox1 and NoxR1 are required for recruitment of polarity cues and actin to prospective fusion sites during trap loop formation. (A–C)** Localization of Chs1-mNG, Tea1-GFP, and Lifeact-GFP in the WT (A), *nox1* (B), and *noxr1* (C) during trap formation from 225 to 300 min after *C. elegans* exposure. Merged images show mNG or GFP fluorescence overlaid with SR2200-stained cell walls. White arrows indicate prospective fusion sites. Scale bars, 20 μm. Images are representative of three independent biological replicates. mpe, minutes post-nematode exposure.

We then investigated whether localized Nox1-dependent ROS signaling directs the recruitment of cell polarity proteins and cytoskeletal elements to the prospective fusion site on the receiving hypha. In the WT, right before the trap hyphae fused with the receiving hyphae, Chs1-mNG, Tea1-GFP, and Lifeact-GFP accumulated intensely not only at the growing trap apex but also at the prospective fusion site on the receiving hyphae by 225 mpe (Fig. 8 A; and Videos 8 and 9). In contrast, these proteins failed to accumulate at the prospective fusion site on receiving hyphae in the *nox* mutants (Fig. 8, B and C; and Videos 8 and 9). Consequently, trap hyphae failed to fuse with the receiving hypha upon contact and instead continued to loop (Fig. 8, B and C; and Videos 8 and 9). Since septin assembly during plant infection in *M. oryzae* depends on Nox activity (Ryder et al., 2013), we examined Sep4 dynamics in the *noxr1* mutant. Sep4-mNG retained its asymmetric localization along the inner rim of trap loop and assembled normally into a ring at the base of infection bulb in the *noxr1* mutant (Fig. S5, E and F; and Video 10).

**Video 8.** **Nox1-mediated ROS signaling directs recruitment of polarity markers to the fusion sites during trap loop closure.** Time-lapse fluorescence video of WT and *nox* mutants expressing Chs1-mNG or Tea1-GFP (green) and costained with SR2200 (white) during trap development following 3 h of nematode exposure. This video corresponds to the micrographs shown in Fig. 8. Images were acquired every 5 min and are displayed at 2 frames per second. The time stamp indicates the time elapsed since exposure to *C. elegans* and is displayed in HH:MM:SS format. Scale bar, 20 μm.

**Video 9.** **Nox1-mediated ROS signaling directs recruitment of F-actin to the fusion sites during trap loop closure.** Time-lapse fluorescence video of WT and *noxr1* mutant expressing Lifeact-GFP (green) and costained with SR2200 (white) during trap development following 3 h of nematode exposure. This video corresponds to the micrographs shown in Fig. 8. Images were acquired every 5 min and are displayed at 2 frames per second. The time stamp indicates the time elapsed since exposure to *C. elegans* and is displayed in HH:MM:SS format. Scale bar, 20 μm.

**Video 10.** **Asymmetric localization of septins along the inner rim is Nox1-independent during trap formation.** Time-lapse fluorescence video of WT and *noxr1* mutants expressing Sep4-mNG (green) and costained with SR2200 (white) during trap development following 3 h of nematode exposure. This video corresponds to the micrographs shown in Fig. S5 E. Images were acquired every 5 min and are displayed at 2 frames per second. The time stamp indicates the time elapsed since exposure to* C. elegans *and is displayed in HH:MM:SS format. Scale bar, 20 μm.

Collectively, these findings identify localized Nox1-dependent ROS as a spatial landmark that specifies the prospective fusion site and promotes hyphal fusion by directing the recruitment of polarity proteins and F-actin, while remaining dispensable for septin organization during trap morphogenesis.

## Discussion

**Figure 9.**
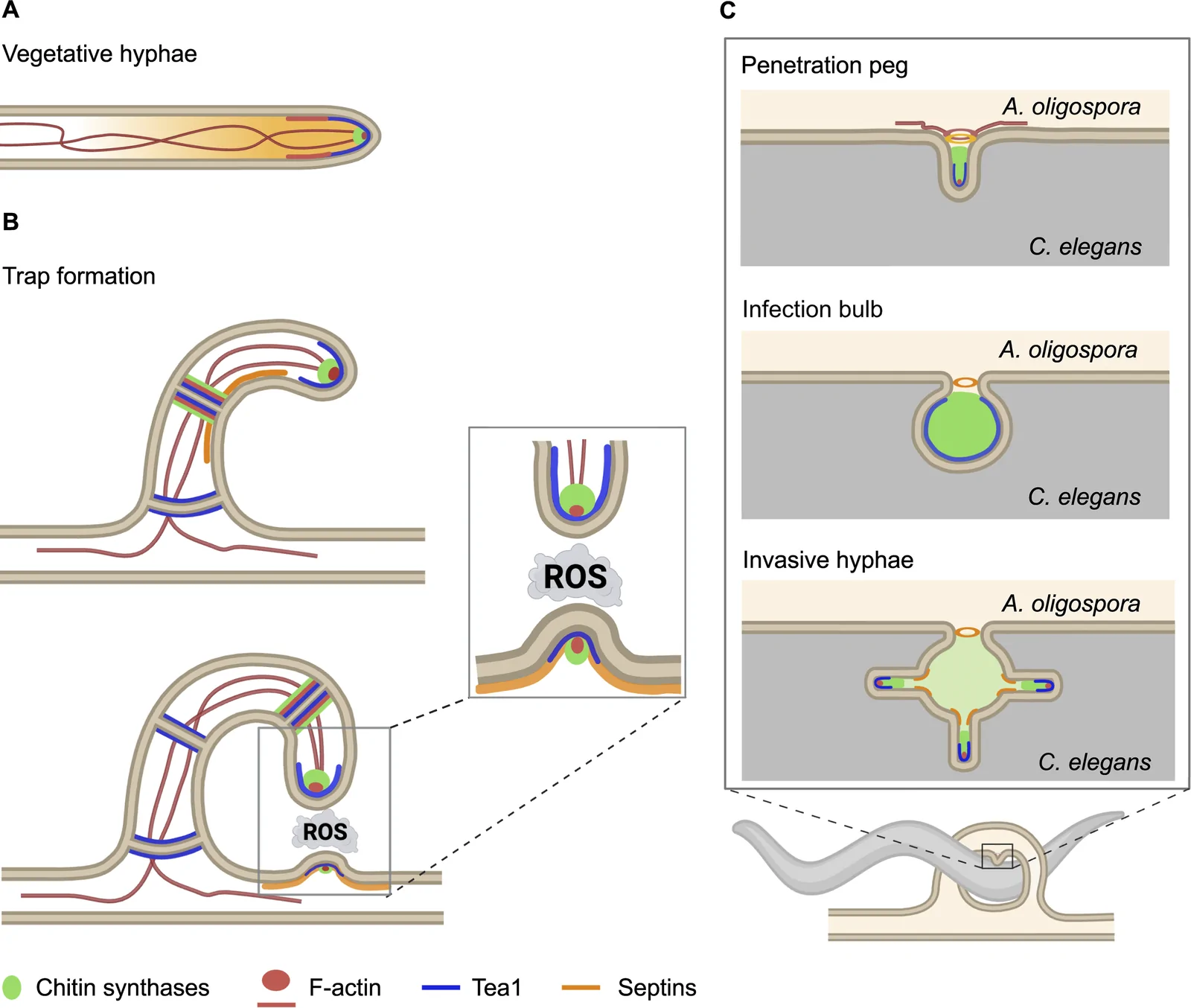
**Hypothetical model of cell polarity cues, cytoskeletal dynamics, and NADPH oxidase signaling during the predatory life cycle of *A. oligospora*. (A)** During vegetative hyphal growth, the CHSs Chs1 and Chs7 (green) localize to the SPK and hyphal apex, respectively. F-actin (red) accumulates at the tip and in subapical patches. Tea1 (blue) marks the hyphal apex, and septins (orange) form a diffuse structure at the hyphal tip while remaining excluded from the SPK. **(B)** During trap loop development, CHSs, Tea1, and F-actin relocalize to growing trap apex, whereas septins asymmetrically localize along the inner rim of the developing loop (top panel). At the prospective fusion site (lower panel), Nox1-dependent ROS production recruits CHSs, Tea1, and F-actin to the receiving hypha to promote cell fusion, whereas septins localize to the branch point at the prospective fusion site. **(C)** Following nematode capture, infection structures develop through penetration peg formation (upper panel), infection bulb isotropic expansion (middle panel), and multipolar invasive hyphal growth (lower panel). F-actin and septins assemble into a ring at the base of the infection bulb, Tea1 accumulates around the bulb, and CHSs become diffusely distributed throughout the bulb. During invasive hyphal growth, F-actin, CHSs, and Tea1 relocalize to invasive hyphal tips, whereas septins accumulate at branch points.

The development of nematode-trapping structures requires repeated reconfiguration of conserved polarity, cytoskeletal, and ROS signaling modules during distinct morphogenetic transitions (Fig. 9). Here, we define these transitions as directional hyphal reorientation, site-specific hyphal fusion, and the switch from isotropic infection bulb expansion to multipolar invasive hyphal growth.

A central morphogenetic transition during trap development is the conversion of linear vegetative hyphal growth into a curved trapping loop (Fig. 9, A and B). Our quantitative analyses indicate that this curvature is generated through repeated localized redirection of hyphal growth rather than intrinsically curved tip extension. Consistent with this model, Tea1 and Chs1 undergo dynamic redistribution to the trap apex during trap loop formation (Fig. 9 B). This mechanism contrasts with the canonical role of Tea1 in maintaining linear polarized growth in *S. pombe* and *A. nidulans* (Mata and Nurse, 1997; Takeshita et al., 2013), indicating that the polarity module has been evolutionarily co-opted to drive repeated growth reorientation during trap morphogenesis in NTF. Tea1 also remains associated with septa throughout development, consistent with observations in another NTF, *A. flagrans* (Kriegler et al., 2025b). Given that the *tea1* mutant remains capable of septum formation, the biological significance of this conserved septal localization remains unclear in NTF.

Redirecting hyphal growth establishes trap geometry, but maintaining this geometry requires extensive cytoskeletal remodeling. While F-actin remains concentrated at growing trap apex, septins asymmetrically localize along the inner rim of the developing trap loop (Fig. 9 B), reflecting the conserved ability of septins to recognize curved membrane domains (Bridges et al., 2016). In addition to sensing membrane curvature, septins associate with phosphoinositides and sphingolipids in other fungi (Casamayor and Snyder, 2003; Mela and Momany, 2021), suggesting that membrane lipid organization may contribute to septin recruitment during trap morphogenesis. Membrane curvature is shaped by the coordinated action of intrinsic membrane properties and membrane-associated proteins that generate local mechanical forces during membrane remodeling (Jarsch et al., 2016). Future application of fluorescence lifetime-based mechanoprobes (Ryder et al., 2023) could determine whether dynamic changes in plasma membrane mechanics accompany trap morphogenesis.

The final step in trap loop morphogenesis is site-specific hyphal fusion. Recent work in *A. flagrans* established that NoxA and NoxR are essential for trap cell fusion and revealed oscillatory localization of NoxR at trap hyphal tips during this process (Kriegler et al., 2026). Our analyses further show that loss of Nox1 or NoxR1 abolishes the recruitment of polarity markers and F-actin to the receiving hypha and support a model in which Nox1-generated ROS establishes a localized cortical signaling domain on the receiving hypha that recruits polarity proteins and F-actin for membrane fusion. In *Neurospora crassa*, the pheromone-responsive MAPK pathway regulates *NOX1* expression (Cano-Domínguez et al., 2008; Leeder et al., 2013). Given that the same MAPK pathway is essential for trap development in *A. oligospora* (Chen et al., 2021), we hypothesize that this pathway similarly regulates Nox1-dependent signaling during trap cell fusion. Identifying downstream ROS-responsive components will further explain how fungal cells specify prospective fusion sites with high spatial precision.

Following nematode capture, the polarity machinery is reorganized again to support the transition from isotropic infection bulb expansion to multipolar invasive growth (Fig. 9 C). During infection bulb formation, polarity proteins initially become broadly distributed throughout the expanding bulb, consistent with isotropic growth, before resolving into multiple discrete polarity sites that seed invasive hyphae. The coordinated assembly of septins and F-actin into a ring during infection bulb formation resembles the septin collars formed in the appressorium of *M. oryzae* (Dagdas et al., 2012). Recent application of fluorescence lifetime-based mechanoprobes revealed dynamic changes in plasma membrane tension during appressorium maturation in *M. oryzae* (Ryder et al., 2023). Given the similar cytoskeletal organization of infection bulbs and appressoria, it will be important to determine whether comparable changes in membrane mechanics accompany infection bulb maturation in *A. oligospora*. Subsequent enrichment of Tea1 and CHSs at *A. oligospora* invasive hyphal tips further reflects the reestablishment of polarized growth as invasive hyphae emerge from the expanding infection bulb. Together, these parallels suggest diverse fungi have converged on conserved cellular strategies to construct specialized infection structures for host invasion.

In summary, our study suggests that complex fungal infection structures can evolve through the redeployment of conserved polarity, cytoskeletal, and ROS signaling modules in various systems rather than through the invention of new morphogenetic mechanisms. The striking mechanistic parallels between NTF and plant pathogens suggest that diverse fungal infection structures have emerged by co-opting a shared repertoire of cellular machinery despite their independent evolutionary origins (Eisermann et al., 2023). As a genetically tractable predator with highly stereotyped developmental transitions, *A. oligospora* provides a powerful model for uncovering how conserved cellular machinery is repurposed to generate novel structures in response to environmental cues.

## Materials and methods

### Strains and culture conditions

Fungal strains were maintained routinely on PDA (Difco). Trap induction and nematode escaping rate assays were all performed on low-nutrient medium agar (LNM: 2% agar, 1.66 mM MgSO_4_, 5.4 µM ZnSO_4_, 2.6 µM MnSO_4_, 18.5 µM FeCl_3_, 13.4 mM KCl, 0.34 µM biotin, and 0.75 µM thiamin). Liquid culture of fungi to acquire protoplasts was conducted in potato dextrose broth (PDB; Difco). *C. elegans* N2 (Bristol) was maintained on standard nematode growth medium with *Escherichia coli* (OP50) as the food source. All fungal strains used are listed in Table S2.

### Bioinformatics analyses of CHSs, cell-end markers, septins, and NOX enzymes in the *A. oligospora* genome

The amino acid sequences of each CHS, cell-end marker homolog, septin family member, and NOX enzyme from *Saccharomyces cerevisiae*, *S. pombe*, *A. nidulans*, *A. flagrans*, *N. crassa*, and *M. oryzae*, and *Fusarium graminearum*, were used as queries to identify their homologs in *A. oligospora* using local BLAST analysis in Blast2GO 5 PRO (Conesa et al., 2005).

To construct phylogenies of CHSs, cell-end markers, septins, and NOX enzymes, multiple sequence alignments of proteins from the same family were generated using the T-Coffee program (Taly et al., 2011). Pairwise sequence identity percentages were calculated from the resulting alignment. Phylogenetic trees were constructed using FastTree (Price et al., 2009) and visualized using iTOL (Letunic and Bork, 2007). Protein domain predication for each gene was generated using InterPro (Blum et al., 2024). A hypothetical model of polarized growth and trap development in *A. oligospora* was illustrated in BioRender (https://biorender.com).

### Construction of fluorescent fusion plasmids

A complete list of plasmids used and generated is presented in Table S2. Fluorescent protein constructs for Lifeact, cell-end markers, CHSs, and septins were generated using GFP or mNG. Briefly, each target gene, along with a 2-kb upstream sequence, was amplified using specific primers (all primer sequences are in Table S3) and cloned into a plasmid containing a Geneticin resistance cassette and encoding the respective fluorescent protein. The resulting plasmids were transformed into TWF154 strain, and transformants carrying fluorescent protein–tagged target genes were examined using fluorescence microscopy.

### Live-cell imaging of fungal development

To assess the localization of CHSs, cell-end markers, F-actin, and septins, strains expressing fluorescent protein fusions were inoculated onto 5.5-cm LNM plates and incubated at 25°C. Vegetative hyphal tips were imaged after 2 days of growth, whereas 3-day-old cultures were utilized as the starting material for subsequent trap induction and infection structure differentiation. For vegetative hyphae, cultures were stained with SCRI Renaissance 2200 (SR2200; #2SCRI24; Renaissance Chemicals) and FM4-64 (#T3166; Invitrogen) to label fungal cell walls and plasma membranes, respectively. Fluorescent protein fusions included GFP and mNG. Imaging was performed at room temperature using an LSM 980 Airyscan 2 confocal microscope (Carl Zeiss) equipped with a Plan-Apochromat 100×/1.46 Oil DIC objective and an Airyscan 2 detector. Images were acquired in Multiplex Mode (SR-4Y) and processed using Zeiss ZEN Blue software and Fiji (Schindelin et al., 2012).

For developmental analysis, *A. oligospora* cultures were exposed to ∼400 *C. elegans* L4-stage larvae per plate to induce trap formation. To monitor trap development, nematodes were removed after 3 h by washing with deionized water. For infection structure analysis, cultures were exposed to nematodes for 24 h. To capture the initial stages of penetration, nematodes were introduced to established traps and incubated for 10 min before imaging. Fungal cell walls were stained with SR2200. For imaging, agar blocks containing the fungal structures were excised from the LNM plates and inverted onto coverslips. Live-cell imaging was performed at 25°C using either a Nikon Ti-E microscope equipped with an Andor Revolution WD spinning-disk system and an iXON Ultra 888 EMCCD camera (Andor) or a Leica DMi8 inverted microscope equipped with a CrestOptics spinning-disk system and an ORCA-Fusion digital CMOS camera (Hamamatsu). Images were acquired using either a CFI Apochromat TIRF 100×/1.49 oil objective (Nikon) or an HC PL APO 63×/1.40 Oil CS2 objective (Leica), with MetaMorph (Nikon/Andor system) or LAS X (Leica system), respectively. Images were processed using MetaMorph, LAS X, and Fiji (Schindelin et al., 2012).

### Image analysis and quantification

For quantitative analyses, fluorescence intensity was measured using Fiji (Schindelin et al., 2012). Trap cell curvature was quantified using the Fiji plugin Kappa, which measures curvature based on cubic B-spline fitting (Mary and Brouhard, 2019, *Preprint*). Individual trap loops containing three cells were divided into proximal, middle, and distal cells for separate analysis. The centerline of each cell was manually traced, and the average curvature (µm^−1^) of the fitted spline was recorded.

Growth direction change was quantified using the Fiji Angle tool. For each cell, defined by two consecutive septa, the initial growth direction was represented by a line perpendicular to the proximal septum, whereas the subsequent growth direction was represented by a line connecting the centers of the proximal and distal septa. Growth direction change (θ) was calculated as the angle between these two lines. Vegetative hyphae and proximal, middle, and distal trap cells were analyzed separately. Measurements from multiple cells were pooled for statistical analysis.

For quantification of septin asymmetry, fluorescence intensity was measured along the inner and outer rims of the entire curved region of the developing trap loop, and the inner-to-outer fluorescence intensity ratio was calculated for each trap.

### Generation of gene deletion mutants and complementation strains

Targeted gene deletions were generated in the *ku70* mutant strain of *A. oligospora* TWF1697, as its inherent NHEJ deficiency boosts the frequency of homologous recombination. In brief, 1.5 kb of the 5′ and -3′ regions flanking the open reading frame (ORF) of the target gene was amplified and fused to a clonNAT resistance cassette (amplified from plasmid pRS41N) or a Geneticin resistance cassette (amplified from plasmid pUMa1057) to generate the knockout constructs. The constructs were then introduced into protoplasts of the *ku70* background by PEG-mediated transformation. To generate protoplasts, two 5.5-cm plates of *ku70* cultured on PDA were inoculated in 50 ml of PDB and incubated for 24 h at 25°C at 200 rpm. Fungal hyphae were collected by centrifugation and washed using MN buffer (0.3 M MgSO_4_, 0.3 M NaCl). Blended mycelia were mixed with 50 mg/ml VinoTaste Pro lytic enzyme in MN buffer overnight at 25°C at 200 rpm. Protoplasts were collected by filtering the mixture through 3 layers of Miracloth and washed with STC buffer (1.2 M sorbitol, 10 mM Tris-HCl, pH 7.5, 50 mM CaCl_2_). Next, 5 × 10^5^ protoplasts were mixed with 3 μg of construct DNA and incubated on ice for 30 min. Five volumes of PTC buffer (40% PEG 4000 [wt/vol], 10 mM Tris-HCl, pH 7.5, 50 mM CaCl_2_) were then added, and the mixture was incubated for 20 min at room temperature. Then, molten regeneration agar (1% agar, 0.5 M sucrose, 0.3% yeast extract [wt/vol], 0.3% casein acid hydrolysate [wt/vol]) containing 180 μg/ml clonNAT or 220 μg/ml Geneticin was added to the protoplasts and poured onto petri dishes. Transformants were then screened for gene deletion. Primers used for gene deletion and genotyping are listed in Table S3.

Successful knockouts of target genes were first confirmed by PCR, and then validated by Southern blotting (Fig. S4 A) to establish the presence of the ectopically integrated drug cassette as described before (Chen et al., 2021). In transgenes intended for complementation of deletion strains, a copy of the WT locus containing 1.5–2 kb upstream and 1 kb downstream of the ORF was fused with a Geneticin resistance cassette amplified from plasmid pUMa1057 by PCR and transformed into the protoplasts of targeted mutant strains.

### Phenotypic characterization

Trap formation in response to *C. elegans* was quantified as described previously (Yang et al., 2020). Briefly, fungal strains were grown on 3.5-cm LNM plates at 25°C for 48 h. Subsequently, 30 *C. elegans* L4-stage larvae were introduced onto the fungal plates and incubated for 6 h before removal. After 24 h of nematode exposure, three micrographs within 0.5 cm of the plate edge were captured using a Zeiss Stemi 305 microscope with a Zeiss Axiocam ERc 5s camera at 40× magnification. Trap numbers were quantified from all three images.

To investigate the role of microtubule polymerization in trap morphogenesis, 200 μl of benomyl solution (125 µg/ml in 1.6% DMSO) or solvent control (1.6% DMSO) was added directly onto fungal mycelia grown on 3-cm LNM plates, resulting in a final benomyl concentration of 5 µg/ml (0.06% DMSO). Plates were incubated for 1 h before exposure to *C. elegans*, and trap formation was quantified as described above.

To compare trap morphology and quantify the nematode escaping rate in WT and mutant strains, fungi were inoculated onto 5.5-cm LNM plates and incubated at 25°C for 3 days. Cultures were then exposed to ∼400 *C. elegans* L4-stage larvae per plate for 24 h to induce trap formation. For trap morphology analysis, cultures were stained with SR2200 to label fungal cell walls. Imaging was performed using an LSM 980 Airyscan 2 confocal microscope (Carl Zeiss) equipped with a Plan-Apochromat 40×/1.2 water DIC objective and an Airyscan 2 detector. Images were acquired with Multiplex Mode (SR-4Y) and processed using Zeiss ZEN Blue software.

For the nematode escaping rate quantification, 30 adult nematodes were placed onto established traps and allowed to crawl on the fungal lawn for 10 min. Untrapped nematodes were removed by gently washing the plate with deionized water. The nematode escaping rate was calculated as the proportion of untrapped nematodes relative to the total number of nematodes tested.

### Detection of superoxide during trap development

Superoxide production was detected by staining fungal hyphae with NBT, which is reduced by superoxide to a water-insoluble blue formazan precipitate. Trap induction was performed as described in the previous section. To visualize superoxide, 1 ml of freshly prepared 0.1% (wt/vol) NBT in 0.05 M sodium phosphate buffer was added to each plate and incubated for 20 min at room temperature in the dark. Excess dye was removed by rinsing twice with PBS, and samples were imaged immediately. Imaging was performed on an LSM 980 Airyscan 2 confocal microscope (Carl Zeiss) equipped with a Plan-Apochromat 40×/1.2 water DIC objective and an Airyscan 2 detector. Images were acquired in Multiplex Mode (SR-4Y) and processed with Zeiss ZEN Blue software and Fiji (Schindelin et al., 2012).

### Statistical analysis

Statistical analyses were performed using data from at least three independent biological replicates. Statistical significance was determined using two-tailed unpaired Student’s *t* tests or one-way analysis of variance followed by uncorrected Fisher’s least significant difference multiple-comparisons test, as indicated in the figure legends. Data distributions were assumed to be normal but were not formally tested. Differences were considered statistically significant at P < 0.05.

### Online supplemental material

Table S1 shows gene names and accession numbers of genes analyzed in this study. Table S2 shows the strains and plasmids used in this study. Table S3 shows the primers used in this study. Fig. S1 shows CHSs are evolutionarily conserved and localize to the vegetative hyphal apex in *A. oligospora*. Fig. S2 shows cell-end markers are evolutionarily conserved, and Tea1 and actin exhibit polarized localization in *A. oligospora*. Fig. S3 shows septins are evolutionarily conserved and localize to curved hyphal branch bases in *A. oligospora*. Fig. S4 shows validation of gene deletion mutants and phenotypic analysis of *tea* mutants. Fig. S5 shows Nox1 and NoxR1 are conserved and induced during trap morphogenesis but are dispensable for septin assembly in *A. oligospora*. Video 1 shows dynamic reorganization of F-actin during hyphal extension and septum formation. Video 2 shows cell polarity proteins are recruited to hyphal tip and fusion sites during trap formation. Video 3 shows F-actin accumulates at hyphal tip and fusion sites during trap formation. Video 4 shows septins form an asymmetric inner-rim scaffold during trap formation. Video 5 shows spatiotemporal reorganization of polarity markers during the transition from the infection bulb to invasive hyphae. Video 6 shows F-actin dynamics during infection structure maturation and invasive growth. Video 7 shows septin dynamics during infection structure maturation and invasive growth. Video 8 shows Nox1-mediated ROS signaling directs recruitment of polarity markers to the fusion sites during trap loop closure. Video 9 shows Nox1-mediated ROS signaling directs recruitment of F-actin to the fusion sites during trap loop closure. Video 10 shows asymmetric localization of septins along inner rim is Nox1-independent during trap formation. Data S1 shows the statistical source data underlying the growth direction change and curvature analyses presented in Fig. 1, B and C. Data S2 shows the measurements underlying the fluorescence intensity, fusion distance, and fusion timing analyses presented in Fig. 2, F–H. Data S3 shows the measurements underlying the infection bulb expansion and hyphal tip growth analyses presented in Fig. 3, B–D. Data S4 shows the measurements and statistical source data underlying the trap formation and nematode escaping rate presented in Fig. 5, C, D, F, and G. Data S5 shows the measurements and statistical source data underlying the trap formation and nematode escaping rate presented in Fig. 6, D–F. Data S6 shows the measurements and statistical source data underlying the trap formation in Fig. 7, C and D. Data S7 shows the sequence identity values underlying Fig. S1, B and C and the TPM values underlying the gene expression analysis in Fig. S1 D. Data S8 shows the TPM values underlying the gene expression analysis in Fig. S2 F. Data S9 shows the sequence identity values underlying Fig. S3 B and the TPM values underlying the gene expression analysis in Fig. S3 C. Data S10 shows the TPM values underlying the gene expression analysis in Fig. S5 D.

## Supplementary Material

Review History

Table S1 shows gene names and accession numbers of genes analyzed in this study.

Table S2 shows the strains and plasmids used in this study.

Table S3 shows the primers used in this study.

Data S1 shows the statistical source data underlying the growth direction change and curvature analyses presented in Fig. 1, B and C.

Data S2 shows the measurements underlying the fluorescence intensity, fusion distance, and fusion timing analyses presented in Fig. 2, F–H.

Data S3 shows the measurements underlying the infection bulb expansion and hyphal tip growth analyses presented in Fig. 3, B–D.

Data S4 shows the measurements and statistical source data underlying the trap formation and nematode escaping rate presented in Fig. 5, C, D, F, and G.

Data S5 shows the measurements and statistical source data underlying the trap formation and nematode escaping rate presented in Fig. 6, D–F.

Data S6 shows the measurements and statistical source data underlying the trap formation in Fig. 7, C and D.

Data S7 shows the sequence identity values underlying Fig. S1, B and C and the TPM values underlying the gene expression analysis in Fig. S1 D.

Data S8 shows the TPM values underlying the gene expression analysis in Fig. S2 F.

Data S9 shows the sequence identity values underlying Fig. S3 B and the TPM values underlying the gene expression analysis in Fig. S3 C.

Data S10 shows the TPM values underlying the gene expression analysis in Fig. S5 D.

## Data Availability

The *A. oligospora* TWF154 genome sequence used in this study is available from NCBI under the accession number GCA_004768765.1. RNA-seq data used in this study are available from the NCBI Gene Expression Omnibus under the accession number GSE233568. All other data supporting the findings of this study are available from the corresponding author upon request.

## References

[bib1] Asnacios, A., and O. Hamant. 2012. The mechanics behind cell polarity. Trends Cell Biol. 22:584–591. 10.1016/j.tcb.2012.08.005 22980034

[bib2] Behrens, R., and P. Nurse. 2002. Roles of fission yeast tea1p in the localization of polarity factors and in organizing the microtubular cytoskeleton. J. Cell Biol. 157:783–793. 10.1083/jcb.200112027 PMC2173414 12034771

[bib3] Benoit, B., C. Poüs, and A. Baillet. 2023. Septins as membrane influencers: Direct play or in association with other cytoskeleton partners. Front. Cell Dev. Biol. 11:1112319. 10.3389/fcell.2023.1112319 PMC9982393 36875762

[bib4] Berepiki, A., A. Lichius, and N.D. Read. 2011. Actin organization and dynamics in filamentous fungi. Nat. Rev. Microbiol. 9:876–887. 10.1038/nrmicro2666 22048737

[bib5] Berepiki, A., A. Lichius, J.-Y. Shoji, J. Tilsner, and N.D. Read. 2010. F-Actin dynamics in *Neurospora crassa*. Eukaryot. Cell. 9:547–557. 10.1128/EC.00253-09 PMC2863416 20139238

[bib6] Blum, M., A. Andreeva, L.C. Florentino, S.R. Chuguransky, T. Grego, E. Hobbs, B.L. Pinto, A. Orr, T. Paysan-Lafosse, I. Ponamareva, . 2024. InterPro: The protein sequence classification resource in 2025. Nucleic Acids Res. 53:D444–D456. 10.1093/nar/gkae1082 PMC11701551 39565202

[bib7] Boyce, K.J., and A. Andrianopoulos. 2015. Fungal dimorphism: The switch from hyphae to yeast is a specialized morphogenetic adaptation allowing colonization of a host. FEMS Microbiol. Rev. 39:797–811. 10.1093/femsre/fuv035 26253139

[bib8] Bridges, A.A., M.S. Jentzsch, P.W. Oakes, P. Occhipinti, and A.S. Gladfelter. 2016. Micron-scale plasma membrane curvature is recognized by the septin cytoskeleton. J. Cell Biol. 213:23–32. 10.1083/jcb.201512029 PMC4828694 27044896

[bib9] Cano-Domínguez, N., K. Alvarez-Delfín, W. Hansberg, and J. Aguirre. 2008. NADPH oxidases NOX-1 and NOX-2 require the regulatory subunit NOR-1 to control cell differentiation and growth in *Neurospora crassa*. Eukaryot. Cell. 7:1352–1361. 10.1128/EC.00137-08 PMC2519770 18567788

[bib10] Casamayor, A., and M. Snyder. 2003. Molecular dissection of a yeast septin: Distinct domains are required for septin interaction, localization, and function. Mol. Cell. Biol. 23:2762–2777. 10.1128/MCB.23.8.2762-2777.2003 PMC152559 12665577

[bib63] Chee, M.K., and S.B. Haase. 2012. New and redesigned pRS plasmid shuttle vectors for genetic manipulation of *Saccharomyce scerevisiae*. G3 (Bethesda). 2:515–526. 10.1534/g3.111.001917 PMC3362935 22670222

[bib11] Chen, S.-A., H.-C. Lin, and Y.-P. Hsueh. 2022. The cAMP-PKA pathway regulates prey sensing and trap morphogenesis in the nematode-trapping fungus *Arthrobotrys oligospora*. G3. 12:jkac217. 10.1093/g3journal/jkac217 PMC9526039 35993904

[bib12] Chen, S.-A., H.-C. Lin, F.C. Schroeder, and Y.-P. Hsueh. 2021. Prey sensing and response in a nematode-trapping fungus is governed by the MAPK pheromone response pathway. Genetics. 217:iyaa008. 10.1093/genetics/iyaa008 PMC8045724 33724405

[bib13] Chiou, J.-G., M.K. Balasubramanian, and D.J. Lew. 2017. Cell polarity in yeast. Annu. Rev. Cell. Dev. Biol. 33:77–101. 10.1146/annurev-cellbio-100616-060856 PMC5944360 28783960

[bib14] Conesa, A., S. Götz, J.M. García-Gómez, J. Terol, M. Talón, and M. Robles. 2005. Blast2GO: A universal tool for annotation, visualization and analysis in functional genomics research. Bioinformatics. 21:3674–3676. 10.1093/bioinformatics/bti610 16081474

[bib15] Crampin, H., K. Finley, M. Gerami-Nejad, H. Court, C. Gale, J. Berman, and P. Sudbery. 2005. *Candida albicans* hyphae have a spitzenkörper that is distinct from the polarisome found in yeast and pseudohyphae. J. Cell Sci. 118:2935–2947. 10.1242/jcs.02414 15976451

[bib16] Dagdas, Y.F., K. Yoshino, G. Dagdas, L.S. Ryder, E. Bielska, G. Steinberg, and N.J. Talbot. 2012. Septin-mediated plant cell invasion by the rice blast fungus, *Magnaporthe oryzae*. Science. 336:1590–1595. 10.1126/science.1222934 22723425

[bib17] Daskalov, A., J. Heller, S. Herzog, A. Fleißner, and N.L. Glass. 2017. Molecular mechanisms regulating cell fusion and heterokaryon formation in filamentous fungi. Microbiol. Spectr. 5:FUNK-0015-2016. 10.1128/microbiolspec.funk-0015-2016 PMC11687462 28256191

[bib18] Dirschnabel, D.E., M. Nowrousian, N. Cano-Domínguez, J. Aguirre, I. Teichert, and U. Kück. 2014. New insights into the roles of NADPH oxidases in sexual development and ascospore germination in *Sordaria macrospora*. Genetics. 196:729–744. 10.1534/genetics.113.159368 PMC3948803 24407906

[bib19] Eisermann, I., M. Garduño-Rosales, and N.J. Talbot. 2023. The emerging role of septins in fungal pathogenesis. Cytoskeleton. 80:242–253. 10.1002/cm.21765 PMC10952683 37265147

[bib20] Fischer, M.S., and N.L. Glass. 2019. Communicate and fuse: How filamentous fungi establish and maintain an interconnected mycelial network. Front. Microbiol. 10:441411. 10.3389/fmicb.2019.00619 PMC6455062 31001214

[bib21] Giraldo, M.C., Y.F. Dagdas, Y.K. Gupta, T.A. Mentlak, M. Yi, A.L. Martinez-Rocha, H. Saitoh, R. Terauchi, N.J. Talbot, and B. Valent. 2013. Two distinct secretion systems facilitate tissue invasion by the rice blast fungus *Magnaporthe oryzae*. Nat. Commun. 4:1996. 10.1038/ncomms2996 PMC3709508 23774898

[bib22] Hernández-Rodríguez, Y., S. Masuo, D. Johnson, R. Orlando, A. Smith, M. Couto-Rodriguez, and M. Momany. 2014. Distinct septin heteropolymers co-exist during multicellular development in the filamentous fungus *Aspergillus nidulans*. PLoS One. 9:e92819. 10.1371/journal.pone.0092819 PMC3963935 24664283

[bib23] Herzog, S., M.R. Schumann, and A. Fleißner. 2015. Cell fusion in *Neurospora crassa*. Curr. Opin. Microbiol. 28:53–59. 10.1016/j.mib.2015.08.002 26340439

[bib24] Hu, X., D.S. Hoffmann, M. Wang, L. Schuhmacher, M.C. Stroe, B. Schreckenberger, M. Elstner, and R. Fischer. 2024. GprC of the nematode-trapping fungus *Arthrobotrys flagrans* activates mitochondria and reprograms fungal cells for nematode hunting. Nat. Microbiol. 9:1752–1763. 10.1038/s41564-024-01731-9 PMC11222155 38877225

[bib25] Jarsch, I.K., F. Daste, and J.L. Gallop. 2016. Membrane curvature in cell biology: An integration of molecular mechanisms. J. Cell Biol. 214:375–387. 10.1083/jcb.201604003 PMC4987295 27528656

[bib26] Khan, A., M. McQuilken, and A.S. Gladfelter. 2015. Septins and generation of asymmetries in fungal cells. Annu. Rev. Microbiol. 69:487–503. 10.1146/annurev-micro-091014-104250 PMC5257338 26488282

[bib27] Koga, H., H. Terasawa, H. Nunoi, K. Takeshige, F. Inagaki, and H. Sumimoto. 1999. Tetratricopeptide repeat (TPR) motifs of p67(phox) participate in interaction with the small GTPase Rac and activation of the phagocyte NADPH oxidase. J. Biol. Chem. 274:25051–25060. 10.1074/jbc.274.35.25051 10455184

[bib28] Kriegler, M., S. Herrero, and R. Fischer. 2025a. Where to grow and where to go. Fungal Genet. Biol. 178:103983. 10.1016/j.fgb.2025.103983 40187481

[bib29] Kriegler, M., V. Wernet, S. Herrero, and R. Fischer. 2026. Spatiotemporal compartmentalization of NADPH oxidase signaling coordinates cell fusion and predatory behavior in nematode-trapping fungi. mBio. 17:e0144126. 10.1128/mbio.01441-26 PMC13463829 42454936

[bib30] Kriegler, M., V. Wernet, B. Hetzer, S. Herrero, A. Wei, J. Wäckerle, I. Dewein, and R. Fischer. 2025b. Cell-end marker proteins are required for hyphal ring formation and size determination of traps in *Arthrobotrys flagrans*. J. Cell Sci. 138:jcs263744. 10.1242/jcs.263744 40270444

[bib61] Kuo, C.-Y., S.-A. Chen, and Y.-P. Hsueh. 2020. The high osmolarity glycerol (HOG) pathway functions in osmosensing, trap morphogenesis and conidiation of the nematode-trapping fungus *Arthrobotrys oligospora*. J. Fungi (Basel). 6:191. 10.3390/jof6040191 PMC7711997 32992476

[bib31] Kuo, C.-Y., R.J. Tay, H.-C. Lin, S.-C. Juan, G. Vidal-Diez de Ulzurrun, Y.-C. Chang, J. Hoki, F.C. Schroeder, and Y.-P. Hsueh. 2024. The nematode-trapping fungus *Arthrobotrys oligospora* detects prey pheromones via G protein-coupled receptors. Nat. Microbiol. 9:1738–1751. 10.1038/s41564-024-01679-w PMC11724650 38649409

[bib32] Leeder, A.C., W. Jonkers, J. Li, and N.L. Glass. 2013. Early colony establishment in *Neurospora crassa* requires a MAP kinase regulatory network. Genetics. 195:883–898. 10.1534/genetics.113.156984 PMC3813871 24037267

[bib33] Letunic, I., and P. Bork. 2007. Interactive tree of life (iTOL): An online tool for phylogenetic tree display and annotation. Bioinformatics. 23:127–128. 10.1093/bioinformatics/btl529 17050570

[bib34] Li, X., Y.-Q. Kang, Y.-L. Luo, K.-Q. Zhang, C.-G. Zou, and L.-M. Liang. 2017. The NADPH oxidase AoNoxA in *Arthrobotrys oligospora* functions as an initial factor in the infection of *Caenorhabditis elegans*. J. Microbiol. 55:885–891. 10.1007/s12275-017-7169-x 29076070

[bib35] Lin, H.-C., G.V.-D. de Ulzurrun, S.-A. Chen, C.-T. Yang, R.J. Tay, T. Iizuka, T.-Y. Huang, C.-Y. Kuo, A.P. Gonçalves, S.-Y. Lin, . 2023. Key processes required for the different stages of fungal carnivory by a nematode-trapping fungus. PLoS Biol. 21:e3002400. 10.1371/journal.pbio.3002400 PMC10662756 37988381

[bib36] Martin, S.G., W.H. McDonald, J.R. Yates, and F. Chang. 2005. Tea4p links microtubule plus ends with the formin for3p in the establishment of cell polarity. Dev. Cell. 8:479–491. 10.1016/j.devcel.2005.02.008 15809031

[bib37] Mary, H., and G.J. Brouhard. 2019. Kappa (κ): Analysis of curvature in biological image data using B-splines. bioRxiv. 10.1101/852772 (Posted November 25, 2019).

[bib38] Mata, J., and P. Nurse. 1997. tea1 and the microtubular cytoskeleton are important for generating global spatial order within the fission yeast cell. Cell. 89:939–949. 10.1016/s0092-8674(00)80279-2 9200612

[bib39] Mela, A., and M. Momany. 2021. Septins coordinate cell wall integrity and lipid metabolism in a sphingolipid-dependent process. J. Cell Sci. 135:jcs258336. 10.1242/jcs.258336 33912961

[bib40] Mostowy, S., and P. Cossart. 2012. Septins: The fourth component of the cytoskeleton. Nat. Rev. Mol. Cell Biol. 13:183–194. 10.1038/nrm3284 22314400

[bib41] Nordbring-Hertz, B., H.B. Jansson, and A. Tunlid. 2001. Nematophagous fungi. *In* Encyclopedia of Life Sciences. John Wiley and Sons, Ltd, Chichester.

[bib42] Nordzieke, D.E., T.R. Fernandes, M. El Ghalid, D. Turrà, and A. Di Pietro. 2019. NADPH oxidase regulates chemotropic growth of the fungal pathogen *Fusarium oxysporum* towards the host plant. New Phytol. 224:1600–1612. 10.1111/nph.16085 31364172

[bib43] Price, M.N., P.S. Dehal, and A.P. Arkin. 2009. FastTree: Computing large minimum evolution trees with profiles instead of a distance matrix. Mol. Biol. Evol. 26:1641–1650. 10.1093/molbev/msp077 PMC2693737 19377059

[bib44] Read, N.D., A.B. Goryachev, and A. Lichius. 2012. The mechanistic basis of self-fusion between conidial anastomosis tubes during fungal colony initiation. Fungal Biol. Rev. 26:1–11. 10.1016/j.fbr.2012.02.003

[bib45] Riquelme, M., J. Aguirre, S. Bartnicki-García, G.H. Braus, M. Feldbrügge, U. Fleig, W. Hansberg, A. Herrera-Estrella, J. Kämper, U. Kück, . 2018. Fungal morphogenesis, from the polarized growth of hyphae to complex reproduction and infection structures. Microbiol. Mol. Biol. Rev. 82:e00068-17. 10.1128/mmbr.00068-00017 PMC5968459 29643171

[bib46] Riquelme, M., and E. Sánchez-León. 2014. The Spitzenkörper: A choreographer of fungal growth and morphogenesis. Curr. Opin. Microbiol. 20:27–33. 10.1016/j.mib.2014.04.003 24858006

[bib47] Roca, M.G., M. Weichert, U. Siegmund, P. Tudzynski, and A. Fleissner. 2012. Germling fusion via conidial anastomosis tubes in the grey mould Botrytis cinerea requires NADPH oxidase activity. Fungal Biol. 116:379–387. 10.1016/j.funbio.2011.12.007 22385620

[bib48] Ryder, L.S., Y.F. Dagdas, T.A. Mentlak, M.J. Kershaw, C.R. Thornton, M. Schuster, J. Chen, Z. Wang, and N.J. Talbot. 2013. NADPH oxidases regulate septin-mediated cytoskeletal remodeling during plant infection by the rice blast fungus. Proc. Natl. Acad. Sci. USA. 110:3179–3184. 10.1073/pnas.1217470110 PMC3581893 23382235

[bib49] Ryder, L.S., S.G. Lopez, L. Michels, A.B. Eseola, J. Sprakel, W. Ma, and N.J. Talbot. 2023. A molecular mechanosensor for real-time visualization of appressorium membrane tension in *Magnaporthe oryzae*. Nat. Microbiol. 8:1508–1519. 10.1038/s41564-023-01430-x PMC10390335 37474734

[bib50] Ryder, L.S., and N.J. Talbot. 2015. Regulation of appressorium development in pathogenic fungi. Curr. Opin. Plant Biol. 26:8–13. 10.1016/j.pbi.2015.05.013 PMC4781897 26043436

[bib51] Schindelin, J., I. Arganda-Carreras, E. Frise, V. Kaynig, M. Longair, T. Pietzsch, S. Preibisch, C. Rueden, S. Saalfeld, B. Schmid, . 2012. Fiji: An open-source platform for biological-image analysis. Nat. Methods 9:676–682. 10.1038/nmeth.2019 PMC3855844 22743772

[bib52] Shuman, B., and M. Momany. 2022. Septins from protists to people. Front. Cell Dev. Biol. 9:824850. 10.3389/fcell.2021.824850 PMC8801916 35111763

[bib53] Snaith, H.A., and K.E. Sawin. 2003. Fission yeast mod5p regulates polarized growth through anchoring of tea1p at cell tips. Nature. 423:647–651. 10.1038/nature01672 12789340

[bib54] Takemoto, D., S. Kamakura, S. Saikia, Y. Becker, R. Wrenn, A. Tanaka, H. Sumimoto, and B. Scott. 2011. Polarity proteins Bem1 and Cdc24 are components of the filamentous fungal NADPH oxidase complex. Proc. Natl. Acad. Sci. USA. 108:2861–2866. 10.1073/pnas.1017309108 PMC3041104 21282602

[bib55] Takeshita, N., Y. Higashitsuji, S. Konzack, and R. Fischer. 2008. Apical sterol-rich membranes are essential for localizing cell end markers that determine growth directionality in the filamentous fungus *Aspergillus nidulans*. Mol. Biol. Cell. 19:339–351. 10.1091/mbc.e07-06-0523 PMC2174190 18003978

[bib56] Takeshita, N., D. Mania, S. Herrero, Y. Ishitsuka, G.U. Nienhaus, M. Podolski, J. Howard, and R. Fischer. 2013. The cell-end marker TeaA and the microtubule polymerase AlpA contribute to microtubule guidance at the hyphal tip cortex of *Aspergillus nidulans* to provide polarity maintenance. J. Cell Sci. 126:5400–5411. 10.1242/jcs.129841 PMC12598972 24101725

[bib57] Taly, J.-F., C. Magis, G. Bussotti, J.-M. Chang, P. Di Tommaso, I. Erb, J. Espinosa-Carrasco, C. Kemena, and C. Notredame. 2011. Using the T-Coffee package to build multiple sequence alignments of protein, RNA, DNA sequences and 3D structures. Nat. Protoc. 6:1669–1682. 10.1038/nprot.2011.393 21979275

[bib58] Tudzynski, P., J. Heller, and U. Siegmund. 2012. Reactive oxygen species generation in fungal development and pathogenesis. Curr. Opin. Microbiol. 15:653–659. 10.1016/j.mib.2012.10.002 23123514

[bib59] Yang, C.-T., G.V.-D. de Ulzurrun, A.P. Gonçalves, H.-C. Lin, C.-W. Chang, T.-Y. Huang, S.-A. Chen, C.-K. Lai, I.J. Tsai, and F.C. Schroeder. 2020. Natural diversity in the predatory behavior facilitates the establishment of a robust model strain for nematode-trapping fungi. Proc. Natl. Acad. Sci. USA. 117:6762–6770. 10.1073/pnas.1919726117 PMC7104180 32161129

[bib62] Youssar, L., V. Wernet, N. Hensel, X. Yu, H.-G. Hildebrand, B. Schreckenberger, M. Kriegler, B. Hetzer, P. Frankino, A. Dillin, . 2019. Intercellular communication is required for trap formation in the nematode-trapping fungus Duddingtonia flagrans. PLoS Genet. 15:e1008029. 10.1371/journal.pgen.1008029 PMC6453484 30917129

[bib60] Zhu, J., W. Zhang, Y. Fan, W. Deng, L. Zhang, S. Wang, X. Liu, and M. Xiang. 2025. Septin AoCDC11 is involved in trap morphogenesis, conidiation, and vegetative growth in carnivorous *Arthrobotrys oligospora*. Fungal Genet. Biol. 177:103971. 10.1016/j.fgb.2025.103971 40023365

