# Supplementary material for: A nematode-trapping fungus orchestrates polarity cues, septins, and NOX signaling for trap formation
Source: J Cell Biol. 2026 Oct 19;225(12):e202603155. doi: 10.1083/jcb.202603155 (PMC13679648; doi:10.1083/jcb.202603155)
Supplement: Supplementary file 1 [file jcb_202603155_review_history.pdf]

JCB

A nematode-trapping fungus orchestrates polarity cues, septins, and NOX signaling for trap formation

Chih-Yen Kuo, Hung-Che Lin, Yun-Zhen Chou, Sheng-An Chen, Hillel Schwartz, and Yen-Ping Hsueh

Corresponding Author(s): Yen-Ping Hsueh, Max Planck Institute for Biology

Review Timeline:

Submission Date:	2026-03-24
Editorial Decision:	2026-04-28
Revision Received:	2026-08-05
Editorial Decision:	2026-09-08
Revision Received:	2026-09-18

Monitoring Editor: Daniel Lew

Scientific Editor: Dan Simon

Transaction Report:

(Note: With the exception of the correction of typographical or spelling errors that could be a source of ambiguity, letters and reports are not edited. The original formatting of letters and referee reports may not be reflected in this compilation.)

DOI: https://doi.org/10.1083/jcb.202603155

April 28, 2026

Re: JCB manuscript #202603155

Yen-Ping Hsueh
Max Planck Institute for Biology

Dear Dr. Hsueh,

Thank you for submitting your manuscript entitled "A nematode-trapping fungus orchestrates polarity cues, septins, and NOX signaling for trap formation." The manuscript was assessed by expert reviewers, whose comments are appended to this letter. We invite you to submit a revision if you can address the reviewers' key concerns, as outlined here.

As you will see, the reviewers (and editors!) appreciate the novelty and interest of understanding the complex morphogenesis of structures like fungal nematode traps. You have focused on a logical set of candidates, and uncovered interesting phenotypes. In its current form, though, the manuscript is perhaps too diffuse for the reader outside the field to appreciate the main points of interest. The two main issues raised by reviewers 1 and 3 are: 1) The need for more quantification of phenotypes and localizations of interest; and 2) an overly diffuse and descriptive text.

With regard to quantification, the reviewers provide multiple suggestions that you may want to consider. In addition, given your speculations on what the different components actually do, it would be good to focus the quantitative analysis on testing your hypotheses. For example, if septins somehow stabilize the curvature of growing hyphae as you suggest, then it would be good to quantify the curvature of the mutant hyphae to see how they deviate from WT in a more systematic manner.

With regard to the writing, Reviewer 1 provides an excellent specific suggestion to emphasize the data on trap morphogenesis and move other data to the supplement. Reviewer 3 highlights as a "weakness" that the paper is descriptive, but at this stage in our knowledge of the system, descriptive work of good quality seems quite appropriate. More serious is the statement that the text reads as a catalogue of results. Particularly in the Discussion, this could be improved by focusing on the questions of interest (like how the hypha that forms a trap makes the correct planar curvature), with less recapitulation of phenotypes (e.g. I'm not sure that saying Tea1 "specifies the spatial coordinates" for hyphal bending adds much beyond restating the phenotype). Speculating about how your components may contribute to the mechanism is fine, but it was not always clear how you envisage that to work. For example, how is persistence of Tea1 at septa relevant to bending the hypha? How would actin and septins "stabilize curvature"?

While you are revising your manuscript, please also attend to the following editorial points to help expedite the publication of your manuscript. Please direct any editorial questions to the journal office.

GENERAL GUIDELINES:

Text limits: Character count for an Article is < 40,000, not including spaces. Count includes title page, abstract, introduction, results, discussion, and acknowledgments. Count does not include materials and methods, figure legends, references, tables, or supplemental legends.

Figures: Articles may have up to 10 main text figures. Figures must be prepared according to the policies outlined in our Instructions to Authors, under Data Presentation, https://jcb.rupress.org/site/misc/ifora.xhtml. All figures in accepted manuscripts will be screened prior to publication.

Statistical analysis: Error bars on graphic representations of numerical data must be clearly described in the figure legend. The number of independent data points (n) represented in a graph must be indicated in the legend. Please indicate whether 'n' refers to technical or biological replicates (i.e. number of analyzed cells, samples or animals, number of independent experiments). If independent experiments with multiple biological replicates have been performed, we recommend using distribution-reproducibility SuperPlots (please see Lord et al., JCB 2020) to better display the distribution of the entire dataset, and report statistics (such as means, error bars, and P values) that address the reproducibility of the findings.

Statistical methods should be explained in full in the materials and methods. For figures presenting pooled data the statistical measure should be defined in the figure legends. Please also be sure to indicate the statistical tests used in each of your experiments (both in the figure legend itself and in a separate methods section) as well as the parameters of the test (for example, if you ran a t-test, please indicate if it was one- or two-sided, etc.). Also, if you used parametric tests, please indicate if the data distribution was tested for normality (and if so, how). If not, you must state something to the effect that "Data distribution was assumed to be normal but this was not formally tested."

***IMPORTANT: It is JCB policy that if requested, original data images must be made available. Failure to provide original

images upon request will result in unavoidable delays in publication. Please ensure that you have access to all original microscopy and blot data images before submitting your revision.***

Supplemental information: There are strict limits on the allowable amount of supplemental data. Articles may have up to 5 supplemental figures. Up to 10 supplemental videos or flash animations are allowed. A summary of all supplemental material should appear at the end of the Materials and methods section.

Please note that JCB now requires authors to submit Source Data used to generate figures containing gels and Western blots with all revised manuscripts. This Source Data consists of fully uncropped and unprocessed images for each gel/blot displayed in the main and supplemental figures. For assays performed using capillary electrophoresis and/or immunoassay-based detection, authors should instead provide the electropherogram graph(s) for each experiment, plotting fluorescence/chemiluminescence intensity vs. molecular weight/size. Please be sure to provide one Source Data file for each figure gels, blots, and/or capillary electrophoresis assays along with your revised manuscript files. File names for Source Data figures should be alphanumeric without any spaces or special characters (i.e., SourceDataF#, where F# refers to the associated main figure number or SourceDataFS# for those associated with Supplementary figures). For traditional gels and blots, the lanes of the gels/blots should be labeled as they are in the associated figure, the place where cropping was applied should be marked (with a box), and molecular weight/size standards should be labeled wherever possible. For capillary electrophoresis assays, each trace in the graph should be color-coded and labeled to indicate which protein, gene, or sample is being measured (please try to avoid red/green combinations to accommodate our color-blind readers).

Source Data files will be made available to reviewers during evaluation of revised manuscripts and, if your paper is eventually published in JCB, the files will be directly linked to specific figures in the published article.

Source Data Figures should be provided as individual PDF files (one file per figure). Authors should endeavor to retain a minimum resolution of 300 dpi or pixels per inch. Please review our instructions for export from Photoshop, Illustrator, and PowerPoint here: https://rupress.org/jcb/pages/submission-guidelines#revised

The typical timeframe for revisions is three to four months. If you anticipate any difficulties in meeting this aforementioned revision time limit, please contact us and we can work with you to find an appropriate time frame for resubmission. Please note that papers are generally considered through only one revision cycle, so any revised manuscript will likely be either accepted or rejected.

When submitting the revision, please include a cover letter addressing the reviewers' comments point by point. Please also highlight all changes in the text of the manuscript.

We hope that the comments below will prove constructive as your work progresses. We would be happy to discuss them further once you've had a chance to consider the points raised in this letter.

Thank you for this interesting contribution to Journal of Cell Biology. You can contact us at the journal office with any questions at cellbio@rockefeller.edu.

Sincerely,

Daniel Lew, PhD
Monitoring Editor
Journal of Cell Biology

Dan Simon, PhD
Scientific Editor
Journal of Cell Biology

Reviewer #1 (Comments to the Authors (Required)):

This study visualizes a range of proteins involved in hyphal growth and polarity maintenance (SPK: Chs1, actin, Tea1, septin, and Nox1) in a nematode-trapping fungus using GFP, and analyzes their localization during hyphal growth and trap formation. Using gene deletion mutants of the tea1 pathway, septins, and the nox1 pathway, the authors reveal structural abnormalities in trap formation.

Combining localization analyses, mutant phenotypes, and previous findings, the study demonstrates that the Tea1 pathway is required for the formation of trap curvature, septins are involved in maintaining this curvature, and the Nox1 pathway is necessary for the fusion between trap hyphae and the original hyphae. Elucidating the roles of this series of pathways involved in trap formation is of significant value. The methods are solid, and the data appear to be reliable.

As indicated by the title, the central focus is the role of these factors in trap formation; however, due to the large number of components examined, the manuscript lacks a clear focus. Figures 1, 2, and 4 present localization analyses in hyphae and penetration hyphae. While these data are relevant for understanding trap formation, they are somewhat predictable. I recommend consolidating these results or moving part of them to the supplementary data, and placing greater emphasis on deepening the analysis of trap formation.

Specific points
Localization analysis (Fig. 3).
The finding that septins are enriched on the inner side of trap hyphae is important. Please quantify and compare the GFP signal intensity between the inner and outer regions of the hyphae.
It is also an important observation that trap curvature is not formed in the tea1 mutant. Please divide the hyphal tip region of Tea1-GFP into inner and outer sides, and quantify and compare the signal intensities.

Trap formation in gene deletion mutants.
In S. pombe and A. nidulans, Tea1 localization is regulated by microtubules and controls actin/SPK localization and the direction of hyphal growth. During trap formation, treatment with a microtubule polymerization inhibitor may produce a phenotype similar to that of the tea1 mutant.

In the sep3 and sep6 mutants, traps fail to form. Quantification similar to that shown in Fig. 5D,E is required. For proper trap formation, it is likely that the curvature of the trap hyphae must be stably maintained in two dimensions in order to reach the original hypha. In the sep3 and sep6 mutants, the failure to form traps may be due to instability of the curvature structure, causing bending in three dimensions and preventing contact with the original hypha. Could this be analyzed using Z-stack imaging?

In the nox1 and noxr1 mutants, trap structures are formed, but fusion with the original hypha does not occur. Although the number of traps is quantified in Fig. 7G, could you also quantify the frequency of hyphal fusion events?

Reviewer #2 (Comments to the Authors (Required)):

A nematode-trapping fungus orchestrates polarity cues, septins, and Nox signaling for trap formation.

The nematode-trapping fungus Arthrobotrys oligospora undergoes a remarkable morphogenetic process by which it forms hyphal traps to capture its nematode prey, which it then invades and consumes. This involves formation of a specific, curved hyphal branch which then fuses to form a circular trap, which constricts in the presence of a nematode. How this process is regulated and controlled has been poorly understood but the group led by Yen-Ping Hseuh, in particular, have made significant progress in analysing the developmental biology of the process.

This submission is of a very high quality and provides new insight into the cell biology of nematode trap formation. In particular it identifies a role for cell polarity proteins, such as Tea1 in directing hyphal growth to form traps and the functions of septins, which form at sites of membrane curvature and at the base of the infection bulb that mediates invasive fungal growth. These septin-dependent processes, shown here to require Sep3 and Sep6 in particular, mirror those of plant pathogenic fungi. This is important from an evolutionary perspective and identifies the fundamental nature of invasive growth regulation by diverse filamentous fungi. Finally, the role of NADPH oxidase in hyphal fusion to form traps is identified, mirroring the mechanism by which conidial anastomosis tubes develop, which are important in mycelial connectivity. Taken together, this is a significant study and is characterised by well devised experiments, excellent live-cell imaging, quantitative analysis and gene functional studies.

My comments are relatively minor.

I would like to see more information on the relationship between Nox function and septin aggregation. Is the role of Nox1 and NoxR associated only with hyphal fusion during trap formation, or is septin aggregation, particularly at the base of the infection bulb dependent upon Nox activity? I realise this might be difficult to address, given that function precedes invasive growth, but has this been looked at? It might require conditional nox mutants to address properly, but have any studies been carried out? Is septin aggregation in the curved 'pig tails' a Nox-independent process, dependent only on curvature, or does it require Nox? The septin ring in Magnaporthe appressoria for example requires Nox activity to form I believe, so it would be good to understand the exact parallel.
Some panels would benefit from potential controls with other markers that are either genral cortical/membrane markers, such as Pma1, or Spitzenkörper or tip markers, such as Mlc, Spa2 or cdc42. This is only a minor point, but sometimes it would be good to see other localisation patterns observed, distinct from those being demonstrated to be tip or curvature-dependent.
Is there a potential mammalian p22phox equivalent, sometimes called noxD, in A.o.? This has been reported in some fungi like Botrytis cinerea? Is there a Bem1 /p40phox analogue? This information might be useful. There is also a further Nox3/C in some

fungi, like Magnaporthe?
One useful addition to this study (but not necessary for any revision and only mentioned as a line of future work) would be to look at the correlation with membrane tension. This could be carried out with the Flipper/Bodipy rotorimetric dyes now available that could show the correlation of trap formation with membrane tension regulation. In this regard the role of TORC2 in the process is likely to be a valuable line to pursue.

Reviewer #3 (Comments to the Authors (Required)):

The nematode-trapping fungi represent fascinating new model systems for studying novel aspects of fungal morphogenesis and pathogenicity. Recent progress in developing genetic tools such as gene insertion now makes it possible to image fluorescent protein fusions and generate knock-out strains in A. oligospora. This manuscript describes the localization and mutant phenotype of polarity factor orthologues in different fungal structures. The traps are networks of fungal cells that grow in curved shapes and fuse with each other. Orthologues of known fungal polarity factors were identified and localized mostly to growing cell tips and septa, similar to what has been shown previously in other fungi. Notably, tea1 and chs1 mutants have defects in trap formation/ fusion. The work also identified nox genes and ROS as important factors in inducing cell-cell fusion, as has been shown in vegetative growth in other fungi. The manuscript includes many movies, which might represent some of the first time-lapse imaging of trap formation and invasion.

General comments

This is a straightforward description of the localization and phenotypes of orthologues of well known polarity factors. This study is important for this field, as it lays the groundwork for studying morphogenesis of traps and other structures in this fungus. A primary weakness is that the manuscript is "descriptive". The text reads as a catalogue of results. Data are shown primarily as representative images and movies, which are described very briefly in the text. There is a distinct lack of quantitation of the data that might bring important mechanistic insights into trap formation (see below). For example, the work does not provide sufficient new insights into how cells grow with a certain curvature, or how cell-cell fusion is achieved. Another weakness is that in terms of molecules, the manuscript does not add very much beyond what has been shown previously in other fungi. Thus, as currently written, this manuscript may not have the significance to a broad audience or the quantitative/ mechanistic cell biological insights needed for publication in this particular journal.

Specific comments

Quantitation of the data is lacking throughout the figures. Here are some types of questions that might be answered with quantitative analyses:

1. What are the distributions of the various phenotypes (i.e. what percentage of traps show each phenotype)?

2. How do the cellular distributions and fluorescence intensities of the factors vary at the different structures and stages? Are the shapes of the tips different among the structures or change before fusion?

3. A detailed quantitative description of trap formation from the movies would add a lot. Do cells form curved shapes during tip growth, or might they grow straight initially?

4. What are rates of cell tip growth in the different structures and stages? Is tip growth constant, or varies with the cell cycle? Are rates of cell tip growth different in the mutants?

5. In analyzing cell fusion, when does the receiving hyphae start to show response? Is it time or a certain distance of the approaching tip that is critical?

6. When are F-actin and septins located on the inner curvature? The images seem to show that they are only on the inner rim of growing cells. Are these cables coming from the tip, or short structures formed on the curved membrane. How does the curvatures of the inner rim compare with the curvature with septin localizes at branch-base? Do they recognize a common curvature? While septins localize to micron-scale curvature (Bridges et al, 2026), I am not aware of studies that actin recognizes the same curvatures (the endocytic pit is much smaller). I would be cautious about proposing an actin-septin mechanism for generating curved walled cells (line 362), as I do not think it is known how binding to curved membranes may guide tip wall growth to grow into a curve.

7. In the pigtail or curvature defective traps, do the fungal tips continue to grow, or stop at the certain length or number of cells?

8. The manuscript curiously does not mention microtubules at all. Are there any data on the role of microtubules?

9. The images of the infection bodies and fungi growing inside the nematode are striking, but their description in the text and

characterization are very limited. Can there be more characterization of the infection bodies and the properties of the invading hyphae? Is this the first movie of them in the worm? Do they grow faster inside the worm? What are preferred paths inside the worm - near the surface or perhaps through the gut?

Subject: Rebuttal Letter for JCB manuscript #202603155

Dear Dr. Simon and Dr. Lew,

We appreciate the thoughtful feedback provided by the reviewers and the opportunity to revise and resubmit our work. We have carefully gone over the reviewers' comments and suggestions, and addressed these concerns in the revised manuscript. Below, we provide a point-by-point response to the reviewers' comments:

Reviewer #1 (Comments to the Authors (Required)):

This study visualizes a range of proteins involved in hyphal growth and polarity maintenance (SPK: Chs1, actin, Tea1, septin, and Nox1) in a nematode-trapping fungus using GFP, and analyzes their localization during hyphal growth and trap formation. Using gene deletion mutants of the tea1 pathway, septins, and the *nox1* pathway, the authors reveal structural abnormalities in trap formation.

Combining localization analyses, mutant phenotypes, and previous findings, the study demonstrates that the Tea1 pathway is required for the formation of trap curvature, septins are involved in maintaining this curvature, and the Nox1 pathway is necessary for the fusion between trap hyphae and the original hyphae. Elucidating the roles of this series of pathways involved in trap formation is of significant value. The methods are solid, and the data appear to be reliable.

As indicated by the title, the central focus is the role of these factors in trap formation; however, due to the large number of components examined, the manuscript lacks a clear focus. Figures 1, 2, and 4 present localization analyses in hyphae and penetration hyphae. While these data are relevant for understanding trap formation, they are somewhat predictable. I recommend consolidating these results or moving part of them to the supplementary data, and placing greater emphasis on deepening the analysis of trap formation.

Our response to Reviewer #1:
We thank the reviewer for the positive evaluation and constructive suggestions. We revised the manuscript by moving the localization analyses in vegetative hyphae to the Supplemental Information and adding quantitative analyses of trap morphogenesis. We also performed the additional experiments and quantifications suggested by the reviewer.

Specific points

1. Localization analysis (Fig. 3).
   The finding that septins are enriched on the inner side of trap hyphae is important. Please quantify and compare the GFP signal intensity between the inner and outer regions of the hyphae.
   It is also an important observation that trap curvature is not formed in the *tea1* mutant. Please divide the hyphal tip region of Tea1-GFP into inner and outer sides, and quantify and compare the signal intensities.

   We quantified septin fluorescence intensity along the inner and outer rims of trap hyphae. The average fluorescence intensity ratios were 1.95 for Sep3-mNG, 2.89 for Sep4-mNG, and 1.84 for

Sep5-GFP (line 207-209; Fig. 2F). Quantification of Tea1-GFP fluorescence at the inner and outer sides of the trap hyphal tip showed average fluorescence intensity ratio of 0.96, indicating that asymmetric localization is specific to septins We further discovered that if we extend nematode exposure to 48 h, the *tea1* mutant eventually was able to form a curved trap loop (line 272-275; Fig. S4 E).

2. Trap formation in gene deletion mutants.
   In *S. pombe* and *A. nidulans*, Tea1 localization is regulated by microtubules and controls actin/SPK localization and the direction of hyphal growth. During trap formation, treatment with a microtubule polymerization inhibitor may produce a phenotype similar to that of the *tea1* mutant.
   We thank the reviewer for this suggestion. We treated WT cultures with benomyl and observed benomyl impaired the development of traps (line 279-283; Fig. 5, E and F). Benomyl-treated hyphae could only form a single loop, whereas the *tea1* mutant predominantly produced stalk-like structures that failed to close into loops. These results indicate that microtubules contribute to trap morphogenesis but treatment with inhibitor does not fully phenocopy the *tea1* mutant.

3. In the *sep3* and *sep6* mutants, traps fail to form. Quantification similar to that shown in Fig. 5D, E is required. In the *sep3* and *sep6* mutants, traps fail to form. Quantification similar to that shown in Fig. 5D, E is required. For proper trap formation, it is likely that the curvature of the trap hyphae must be stably maintained in two dimensions in order to reach the original hypha. In the *sep3* and *sep6* mutants, the failure to form traps may be due to instability of the curvature structure, causing bending in three dimensions and preventing contact with the original hypha. Could this be analyzed using Z-stack imaging?
   We now have included quantification of trap formation and predatory efficiency of the *sep3* and *spe6* mutants in revised manuscript (Fig. 6, D and F). Both mutants showed severe defects in nematode capture. All images in this study were acquired as Z-stacks. Wild-type traps are three-dimensional trap networks rather than two-dimensional loops. The *sep3* and *sep6* mutants have strong defects in trap formation (Fig. 6B and D). In the very few traps that were observed in the mutants, simple curved trap loops were observed. These results indicate that the trap formation defects in the septin mutants are unlikely due to fusion with the original hyphae.

4. In the *nox1* and *noxr1* mutants, trap structures are formed, but fusion with the original hypha does not occur. Although the number of traps is quantified in Fig. 7G, could you also quantify the frequency of hyphal fusion events?
   We quantified cell fusion during trap formation in wild type, *nox1*, and *noxr1* mutants and found that hyphal fusion occurred 100% (39/39) in WT, and 0% in the *nox1* (0/24) or *noxr1* (0/27) mutants.

Reviewer #2 (Comments to the Authors (Required)):

A nematode-trapping fungus orchestrates polarity cues, septins, and Nox signaling for trap formation.

The nematode-trapping fungus *Arthrobotrys oligospora* undergoes a remarkable morphogenetic process by which it forms hyphal traps to capture its nematode prey, which it then invades and consumes. This involves formation of a specific, curved hyphal branch which then fuses to form a circular trap, which constricts in the presence of a nematode. How this process is regulated and controlled has been poorly understood but the group led by Yen-Ping Hseuh, in particular, have made significant progress in analysing the developmental biology of the process.

This submission is of a very high quality and provides new insight into the cell biology of nematode trap formation. In particular it identifies a role for cell polarity proteins, such as Tea1 in directing hyphal growth to form traps and the functions of septins, which form at sites of membrane curvature and at the base of the infection bulb that mediates invasive fungal growth. These septin-dependent processes, shown here to require Sep3 and Sep6 in particular, mirror those of plant pathogenic fungi. This is important from an evolutionary perspective and identifies the fundamental nature of invasive growth regulation by diverse filamentous fungi. Finally, the role of NADPH oxidase in hyphal fusion to form traps is identified, mirroring the mechanism by which conidial anastomosis tubes develop, which are important in mycelial connectivity. Taken together, this is a significant study and is characterised by well devised experiments, excellent live-cell imaging, quantitative analysis and gene functional studies.

Our response to Reviewer #2:
We thank the reviewer for the positive evaluation of our work and the constructive suggestions. In response, we generated new data demonstrating that septin organization is independent of Nox signaling, added localization analyses of the polarity marker Mlc1 during trap and infection development, expanded the phylogenetic analysis of NADPH oxidase components, and revised the Discussion to highlight membrane tension as an important direction for future research.

My comments are relatively minor.

1. I would like to see more information on the relationship between Nox function and septin aggregation. Is the role of Nox1 and NoxR associated only with hyphal fusion during trap formation, or is septin aggregation, particularly at the base of the infection bulb dependent upon Nox activity? I realise this might be difficult to address, given that function precedes invasive growth, but has this been looked at? It might require conditional *nox* mutants to address properly, but have any studies been carried out? Is septin aggregation in the curved 'pig tails' a Nox-independent process, dependent only on curvature, or does it require Nox? The septin ring in *Magnaporthe appressoria* for example requires Nox activity to form I believe, so it would be good to understand the exact parallel.
   We thank the reviewer for this insightful suggestion. We generated a *noxr1* strain expressing Sep4-mNG to examine whether septin organization depends on Nox activity. We found that Sep4 remained asymmetrically localized to the inner rim of the trap loop and a septin ring still forms at the base of the infection bulb in the *noxr1* mutant. These results indicate that septin assembly in

both traps and infection bulb is independent of Nox signaling. The new data are shown in Fig. S5 E and F (line 352-355) in the revised manuscript.

2. Some panels would benefit from potential controls with other markers that are either genral cortical/membrane markers, such as Pma1, or Spitzenkörper or tip markers, such as Mlc, Spa2 or cdc42. This is only a minor point, but sometimes it would be good to see other localisation patterns observed, distinct from those being demonstrated to be tip or curvature-dependent.
   We thank the reviewer for this suggestion. We generated a new strain expressing the tip marker Mlc1-mNG and examined its localization in vegetative hyphae, trap, and infection structures. Mlc1 localized to the hyphal apex in vegetative and trap hyphae, similar to Chs1 and Chs7. During infection, Mlc1 became diffuse in the infection bulb while remaining enriched at the tip of the invasive hypha. These data have been added to the revised manuscript (line 122-123, line 193-195, and line 236-241; Fig. S1 F, Fig. 1D, and Fig. 3E; Video 2 and Video 5).

3. Is there a potential mammalian p22phox equivalent, sometimes called noxD, in A.o.? This has been reported in some fungi like *Botrytis cinerea*? Is there a Bem1 /p40phox analogue? This information might be useful. There is also a further Nox3/C in some fungi, like *Magnaporthe*?
   We identified a putative NoxD/p22phox-like protein in *A. oligospora* TWF154, EYR41_001623, and a Bem1-like protein, EYR41_003604, which may correspond to a fungal Bem1/p40phox-like homolog. However, we did not identify an additional NoxC/Nox3-like NADPH oxidase in TWF154. We have expanded the phylogenetic analysis to include Nox2 and NoxC/Nox3 homologs in the revised manuscript (line 316-318; Fig. S5, A and B).

4. One useful addition to this study (but not necessary for any revision and only mentioned as a line of future work) would be to look at the correlation with membrane tension. This could be carried out with the Flipper/Bodipy rotorimetric dyes now available that could show the correlation of trap formation with membrane tension regulation. In this regard the role of TORC2 in the process is likely to be a valuable line to pursue.
   We thank the reviewer for this insightful suggestion. Due to the current unavailability of FLIM microscopy in our institute, we could not examine membrane tension dynamics during trap and infection development. We have noted this as an important future research direction in the Discussion (line 386-390 and line 409-414).

Reviewer #3 (Comments to the Authors (Required)):

The nematode-trapping fungi represent fascinating new model systems for studying novel aspects of fungal morphogenesis and pathogenicity. Recent progress in developing genetic tools such as gene insertion now makes it possible to image fluorescent protein fusions and generate knock-out strains in *A. oligospora*. This manuscript describes the localization and mutant phenotype of polarity factor orthologues in different fungal structures. The traps are networks of fungal cells that grow in curved shapes and fuse with each other. Orthologues of known fungal polarity factors were identified and localized mostly to growing cell tips and septa, similar to what has been shown previously in other fungi. Notably, *tea1* and *chs1* mutants have defects in trap formation/ fusion. The work also identified nox genes and ROS as important factors in inducing cell-cell fusion, as has been shown in vegetative growth in other fungi. The manuscript includes many movies, which might represent some of the first time-lapse imaging of trap formation and invasion.

General comments:
This is a straightforward description of the localization and phenotypes of orthologues of well known polarity factors. This study is important for this field, as it lays the groundwork for studying morphogenesis of traps and other structures in this fungus. A primary weakness is that the manuscript is "descriptive". The text reads as a catalogue of results. Data are shown primarily as representative images and movies, which are described very briefly in the text. There is a distinct lack of quantitation of the data that might bring important mechanistic insights into trap formation (see below). For example, the work does not provide sufficient new insights into how cells grow with a certain curvature, or how cell-cell fusion is achieved. Another weakness is that in terms of molecules, the manuscript does not add very much beyond what has been shown previously in other fungi. Thus, as currently written, this manuscript may not have the significance to a broad audience or the quantitative/ mechanistic cell biological insights needed for publication in this particular journal.

Specific comments
Quantitation of the data is lacking throughout the figures. Here are some types of questions that might be answered with quantitative analyses:

Our response to Reviewer #3:
We thank the reviewer for the constructive comments. We have substantially expanded the quantitative analyses throughout the manuscript, including trap morphology, trap development, trap cell fusion, infection bulb development, invasive hyphal growth, and fluorescence localization. These new analyses strengthen the mechanistic conclusions of the study.

1. What are the distributions of the various phenotypes (i.e. what percentage of traps show each phenotype)?
   We classified trap morphologies into four categories (network, unfused loop(s), simple loop(s), and branch) and quantified their distributions in the indicated strains (line 268-272, line 303-305, and line 332-334; Fig. 5D, 6E, and 7D). The results show that each mutant exhibits a characteristic trap morphology, supporting distinct roles of cell polarity proteins, septins, and Nox signaling during trap morphogenesis.

2. How do the cellular distributions and fluorescence intensities of the factors vary at the different structures and stages? Are the shapes of the tips different among the structures or change before fusion?

   As described in our response to Reviewer #1, we quantified septin fluorescence intensity along the inner and outer rims of trap hyphae. This analysis demonstrated asymmetric septin enrichment at the inner rim. Besides, we did not observe consistent differences in hyphal tip morphology among the different developmental stages or immediately before trap fusion.

3. A detailed quantitative description of trap formation from the movies would add a lot. Do cells form curved shapes during tip growth, or might they grow straight initially?

   We thank the reviewer for this suggestion. We performed quantitative analyses of trap development by measuring changes in growth direction and cell curvature during trap morphogenesis (lines 179-186; Fig. 1B and C). Compared with vegetative hyphae, each trap cell exhibited changes in growth direction and curvature, with the greatest changes occurring in the middle trap cell. These analyses show that trap curvature is established progressively during trap development.

4. What are rates of cell tip growth in the different structures and stages? Is tip growth constant, or varies with the cell cycle? Are rates of cell tip growth different in the mutants?

   We quantified tip growth rates during vegetative growth, trap development, and invasive growth in the WT strain. The vegetative hyphae grew at ~ 3.8 μm min^{-1}, whereas trap and invasive hyphae elongated at approximately 0.6 and 0.7 μm min^{-1}, respectively (lines 231-233; Fig. 3D). Tip elongation remained relatively constant within each developmental stage. In the mutant strains (*chs1*, *tea1*, *sep3*, *sep6*, *nox1*, and *noxr1*), hyphal tip extension was visibly slower than in the wild type, as evidenced by their reduced colony growth on PDA (Fig. 5A, 6A, and 7B).

5. In analyzing cell fusion, when does the receiving hyphae start to show response? Is it time or a certain distance of the approaching tip that is critical?

   We quantified the onset of the receiving hyphal response using Mlc1 and Sep3 as markers. Recruitment of both proteins began when the approaching hyphal tip was approximately 4 μm away, and trap cell fusion was completed about 15 min later (line 214-218; Fig. 2, E, G and H). Since individual traps are not identical in developmental timing, we conclude that the distance between the approaching hyphal tip and the receiving hypha is a more reliable indicator of the onset of the receiving hyphal response than elapsed time.

6. When are F-actin and septins located on the inner curvature? The images seem to show that they are only on the inner rim of growing cells. Are these cables coming from the tip, or short structures formed on the curved membrane. How does the curvatures of the inner rim compare with the curvature with septin localizes at branch-base? Do they recognize a common curvature? While septins localize to micron-scale curvature (Bridges et al, 2026), I am not aware of studies that actin recognizes the same curvatures (the endocytic pit is much smaller). I would be cautious about proposing an actin-septin mechanism for generating curved walled cells (line 362), as I do not think it is known how binding to curved membranes may guide tip wall growth to grow into a curve.

We thank the reviewer for these insightful comments. We observed that septins began to accumulate along the inner rim during elongation of the middle trap cell as the trapping loop developed. We did not observe septin structures extending from the hyphal tip to the inner rim. In contrast, actin cables remained continuously associated with the growing hyphal tip throughout trap development, indicating distinct localization patterns of the actin and septin cytoskeletons. We also measured the average curvature of the inner trap rim and vegetative branch bases, which were approximately 0.07 and 0.14 μm^{-1}, respectively, indicating that septins localize to membrane regions with a range of curvatures.

We agree that our current data do not provide sufficient evidence to support an actin-septin mechanism underlying trap curvature. Therefore, we removed this speculative model proposing an actin-septin mechanism for generating curved cell walls.

7. In the pigtail or curvature defective traps, do the fungal tips continue to grow, or stop at the certain length or number of cells?
   We found that trap hyphae remained actively growing in both *nox* and *tea1* mutants despite defects in trap development. The *nox* mutant trap hyphae continued to elongate without undergoing hyphal fusion, whereas *tea1* trap hyphae continued to elongate and formed simple trap loops after 48 h of nematode exposure. The new *tea1* data have been added to the revised manuscript (line 272-275; Fig. S4 E).

8. The manuscript curiously does not mention microtubules at all. Are there any data on the role of microtubules?
   As described in our response to Reviewer #1, we examined the role of microtubules by treating wild-type cultures with benomyl. Benomyl markedly reduced trap formation, although treated hyphae still formed simple trap loops (line 279-283; Fig. 5, E and F).

9. The images of the infection bodies and fungi growing inside the nematode are striking, but their description in the text and characterization are very limited. Can there be more characterization of the infection bodies and the properties of the invading hyphae? Is this the first movie of them in the worm? Do they grow faster inside the worm? What are preferred paths inside the worm - near the surface or perhaps through the gut?
   We thank the reviewer for the suggestions. We further characterized infection development by quantifying infection bulb expansion and invasive hyphal growth. Infection bulbs reached an average area of approximately 82 μm^2 after 55 min and expanded at an average rate of 3.3 μm^2 min^{-1} (line 229-231; Fig. 3, B and C). Invasive hyphae elongated at approximately 0.7 μm min^{-1} (line 231; Fig. 3D). To our knowledge, these are the first time-lapse movies capturing infection bulb development and invasive hyphal growth inside living nematodes. Besides, we did not observe a preference for invasive hyphae to grow within specific nematode tissues.

September 8, 2026

RE: JCB Manuscript #202603155R

Yen-Ping Hsueh
Max Planck Institute for Biology

Dear Dr. Hsueh,

Thank you for submitting your revised manuscript entitled "A nematode-trapping fungus orchestrates polarity cues, septins, and NOX signaling for trap formation." We would be happy to publish your paper in JCB pending final revisions necessary to meet our formatting guidelines (see details below). Please also revise the text to address the remaining comments from Reviewer #3.

To avoid unnecessary delays in the acceptance and publication of your paper, please read the following information carefully.

A. MANUSCRIPT ORGANIZATION AND FORMATTING:

Full guidelines are available on our Instructions for Authors page, https://jcb.rupress.org/submission-guidelines#revised.
Submission of a paper that does not conform to JCB guidelines will delay the acceptance of your manuscript.

1) Text limits: Character count for Articles is < 40,000, not including spaces. Count includes title page, abstract, introduction, results, discussion, and acknowledgments. Count does not include materials and methods, figure legends, references, tables, or supplemental legends.

2) Figure formatting: Articles may have up to 10 main text figures. Scale bars must be present on all microscopy images, including inset magnifications. Molecular weight or nucleic acid size markers must be included on all gel electrophoresis. Also, please avoid pairing red and green for images and graphs to ensure legibility for color-blind readers. If red and green are paired for images, please ensure that the particular red and green hues used in micrographs are distinctive with any of the colorblind types. If not, please modify colors accordingly or provide separate images of the individual channels.

3) Statistical analysis: Error bars on graphic representations of numerical data must be clearly described in the figure legend. The number of independent data points (n) represented in a graph must be indicated in the legend. Please indicate whether 'n' refers to technical or biological replicates (i.e. number of analyzed cells, samples or animals, number of independent experiments). If independent experiments with multiple biological replicates have been performed, we recommend using distribution-reproducibility SuperPlots (please see Lord et al., JCB 2020) to better display the distribution of the entire dataset, and report statistics (such as means, error bars, and P values) that address the reproducibility of the findings.

Statistical methods should be explained in full in the materials and methods. For figures presenting pooled data the statistical measure should be defined in the figure legends. Please also be sure to indicate the statistical tests used in each of your experiments (both in the figure legend itself and in a separate methods section) as well as the parameters of the test (for example, if you ran a t-test, please indicate if it was one- or two-sided, etc.). Also, if you used parametric tests, please indicate if the data distribution was tested for normality (and if so, how). If not, you must state something to the effect that "Data distribution was assumed to be normal but this was not formally tested."

4) Materials and methods: Should be comprehensive and not simply reference a previous publication for details on how an experiment was performed. Please provide full descriptions (at least in brief) in the text for readers who may not have access to referenced manuscripts. The text should not refer to methods "...as previously described."

5) For all cell lines, vectors, strains, constructs/cDNAs, etc. - all genetic material: please include database / vendor ID (e.g. Addgene, ATCC, etc.) or if unavailable, please briefly describe their basic genetic features, even if described in other published work or gifted to you by other investigators (and provide references where appropriate). Please be sure to provide the sequences for all of your oligos: primers, si/shRNA, RNAi, gRNAs, etc. in the materials and methods. You must also indicate in the methods the source, species, and catalog numbers/vendor identifiers (where appropriate) for all of your antibodies, including secondary. If antibodies are not commercial, please add a reference citation if possible.

6) Microscope image acquisition: The following information must be provided about the acquisition and processing of images:
a. Make and model of microscope
b. Type, magnification, and numerical aperture of the objective lenses
c. Temperature
d. Imaging medium
e. Fluorochromes
f. Camera make and model

g. Acquisition software
h. Any software used for image processing subsequent to data acquisition. Please include details and types of operations involved (e.g., type of deconvolution, 3D reconstitutions, surface or volume rendering, gamma adjustments, etc.).

7) References: There is no limit to the number of references cited in a manuscript. References should be cited parenthetically in the text by author and year of publication. Abbreviate the names of journals according to PubMed.

8) Supplemental materials: Articles may have up to 5 supplemental figures and 10 videos. Please also note that tables, like figures, should be provided as individual, editable files. A summary of all supplemental material should appear at the end of the Materials and methods section. Please include one brief sentence per item.

9) Video legends: Should describe what is being shown, the cell type or tissue being viewed (including relevant cell treatments, concentration and duration, or transfection), the imaging method (e.g., time-lapse epifluorescence microscopy), what each color represents, how often frames were collected, the frames/second display rate, and the number of any figure that has related video stills or images.

10) eTOC summary: A ~40-50 word summary that describes the context and significance of the findings for a general readership should be included on the title page. The statement should be written in the present tense and refer to the work in the third person. It should begin with "First author name(s) et al..." to match our preferred style.

11) Conflict of interest statement: JCB requires inclusion of a statement in the acknowledgements regarding competing financial interests. If no competing financial interests exist, please include the following statement: "The authors declare no competing financial interests." If competing interests are declared, please follow your statement of these competing interests with the following statement: "The authors declare no further competing financial interests."

12) A separate author contribution section is required following the Acknowledgments in all research manuscripts. All authors should be mentioned and designated by their first and middle initials and full surnames. We encourage use of the CRediT nomenclature (https://casrai.org/credit/).

13) ORCID IDs: ORCID IDs are unique identifiers allowing researchers to create a record of their various scholarly contributions in a single place. Please note that ORCID IDs are required for all authors. At resubmission of your final files, please be sure to provide your ORCID ID and those of all co-authors.

14) JCB requires authors to submit Source Data used to generate figures containing gels and blots (Western, Southern, Northern) with all revised manuscripts. This Source Data consists of fully uncropped and unprocessed images for each gel/blot displayed in the main and supplemental figures. For assays performed using capillary electrophoresis and/or immunoassay-based detection, authors should instead provide the electropherogram graph(s) for each experiment, plotting fluorescence/chemiluminescence intensity vs. molecular weight/size. Since your paper includes cropped gel and/or blot images, please be sure to provide one Source Data file for each figure gels, blots, and/or capillary electrophoresis assays along with your revised manuscript files. File names for Source Data figures should be alphanumeric without any spaces or special characters (i.e., SourceDataF#, where F# refers to the associated main figure number or SourceDataFS# for those associated with Supplementary figures). For traditional gels and blots, the lanes of the gels/blots should be labeled as they are in the associated figure, the place where cropping was applied should be marked (with a box), and molecular weight/size standards should be labeled wherever possible. For capillary electrophoresis assays, each trace in the graph should be color-coded and labeled to indicate which protein, gene, or sample is being measured (please try to avoid red/green combinations to accommodate our color-blind readers).

Source Data files will be directly linked to specific figures in the published article. Source Data Figures should be provided as individual PDF files (one file per figure). Authors should endeavor to retain a minimum resolution of 300 dpi or pixels per inch. Please review our instructions for export from Photoshop, Illustrator, and PowerPoint here: https://rupress.org/jcb/pages/submission-guidelines#revised.

15) Journal of Cell Biology now requires a data availability statement for all research article submissions. These statements will be published in the article directly above the Acknowledgments. The statement should address all data underlying the research presented in the manuscript. Please visit the JCB instructions for authors for guidelines and examples of statements at (https://rupress.org/jcb/pages/editorial-policies#data-availability-statement).

B. FINAL FILES:

Please upload the following materials to our online submission system. These items are required prior to acceptance. If you have any questions, contact JCB's Managing Editor, Lindsey Hollander (lhollander@rockefeller.edu).

-- An editable version of the final text (.DOC or .DOCX) is needed for copyediting (no PDFs).

-- High-resolution figure and MP4 video files: See our detailed guidelines for preparing your production-ready images, https://jcb.rupress.org/fig-vid-guidelines.

-- Cover images: If you have any striking images related to this story, we would be happy to consider them for inclusion on the journal cover. Submitted images may also be chosen for highlighting on the journal table of contents or JCB homepage carousel. Images should be uploaded as TIFF or EPS files and must be at least 300 dpi resolution.

It is JCB policy that if requested, original data images must be made available to the editors. Failure to provide original images upon request will result in unavoidable delays in publication. Please ensure that you have access to all original data images prior to final submission.

The license to publish form must be signed before your manuscript can be sent to production. A link to the license to publish form will be sent to the corresponding author only. Please take a moment to check your funder requirements before choosing the appropriate license.

Additionally, JCB encourages authors to submit a short video summary of their work. These videos are intended to convey the main messages of the study to a non-specialist, scientific audience. Think of them as an extended version of your abstract, or a short poster presentation. We encourage first authors to present the results to increase their visibility. The videos will be shared on social media to promote your work. For more detailed guidelines and tips on preparing your video, please visit https://rupress.org/jcb/pages/submission-guidelines#videoSummaries.

Thank you for your attention to these final processing requirements. Please revise and format the manuscript and upload materials within 14 days. If you need an extension for whatever reason, please let us know and we can work with you to determine a suitable revision period.

Please contact the journal office with any questions at cellbio@rockefeller.edu.

Thank you for this interesting contribution, we look forward to publishing your paper in Journal of Cell Biology.

Sincerely,

Daniel Lew, PhD
Monitoring Editor
Journal of Cell Biology

Dan Simon, PhD
Scientific Editor
Journal of Cell Biology

Reviewer #1 (Comments to the Authors (Required)):

I have confirmed that the manuscript has been appropriately revised.

Reviewer #2 (Comments to the Authors (Required)):

I am grateful to the authors for responding constructively to all of the comments I made regarding the initial submission. The authors have addressed the comments of all three reviewers very well and I think the revised manuscript is much improved as a consequence. I have no further comments and happy to recommend publication.

Reviewer #3 (Comments to the Authors (Required)):

In this revision, the manuscript has been improved by addition of ample new data and in improvements in the writing. This reviewer appreciates the new quantitative data which enriches the work and addresses questions from me and the other reviewers. Importantly, the rewriting of the text provides more context of the results and a much-improved narrative. I have only minor comments to be addressed before acceptance for publication.

In Figure 2F, the text cannot conclude that septin intensity is approximately "2-fold higher". The data (from very low sample sizes) show that a fraction of cells has a ratio close to 1, and then a subset with range of ratios of 2.5 to 4.5. There are no clusters of cells with ratio near 2. Thus, presenting a mean with SD is not appropriate for this non-Gaussian distribution. A more accurate description would be something like: "20-50% of cells showed ratio of 2.5 or more, with little difference in the remainder of the cells". A larger sample size would certainly make these measurements more robust, especially when only a small subset

is showing the phenotype. In general, the authors should reexamine the statistical tests and confirm that description of the data is appropriate. In addition, can the authors provide a more thorough description of the ROIs that intensities were measured in? Unlike the schematic Fig. 2E, the images suggest that the asymmetric septin enrichment is mainly in the growing tip cell, and not in the whole structure.

Statistics. The meaning of "Different letters indicate significant differences" in legends is not clear. Can the authors clarify please?

<u>2nd Revision - Authors' Response to Reviewers: September 18, 2026</u>

Subject: Rebuttal Letter for JCB manuscript #202603155

Dear Dr. Simon and Dr. Lew,

We are pleased to learn that Reviewer #1 and #2 like the revised manuscript. In response to reviewer #3's comments, we have carefully addressed the points in the revised manuscript.

Thank you again for all the constructive feedbacks to improve this work.

<u>Reviewer #3 (Comments to the Authors (Required)):</u>

In this revision, the manuscript has been improved by addition of ample new data and in improvements in the writing. This reviewer appreciates the new quantitative data which enriches the work and addresses questions from me and the other reviewers. Importantly, the rewriting of the text provides more context of the results and a much-improved narrative. I have only minor comments to be addressed before acceptance for publication.

1. In Figure 2F, the text cannot conclude that septin intensity is approximately "2-fold higher". The data (from very low sample sizes) show that a fraction of cells has a ratio close to 1, and then a subset with range of ratios of 2.5 to 4.5. There are no clusters of cells with ratio near 2. Thus, presenting a mean with SD is not appropriate for this non-Gaussian distribution. A more accurate description would be something like: "20-50% of cells showed ratio of 2.5 or more, with little difference in the remainder of the cells". A larger sample size would certainly make these measurements more robust, especially when only a small subset is showing the phenotype. In general, the authors should reexamine the statistical tests and confirm that description of the data is appropriate. In addition, can the authors provide a more thorough description of the ROIs that intensities were measured in? Unlike the schematic Fig. 2E, the images suggest that the asymmetric septin enrichment is mainly in the growing tip cell, and not in the whole structure.
   We thank the reviewer for raising this point. We have clarified the description of the fluorescence measurements in the figure legend and Methods. The ROIs encompassed the entire curved region of the developing trap, including the inner concave rim and corresponding outer rim, to quantify septin distribution along the curved structure rather than being restricted to the growing tip. We have also revised the text to describe the quantitative results more accurately and removed the statement that septin intensity is approximately twofold higher. Instead, we now report that approximately 20–50% of traps exhibited an inner-to-outer fluorescence intensity ratio >2, whereas the remaining cells showed relatively little difference between the two sides (line 206-209).

2. Statistics. The meaning of "Different letters indicate significant differences" in legends is not clear. Can the authors clarify please?
   We have clarified the description of the statistical analysis in the figure legends. Specifically, we now state that groups labeled with different letters are significantly different, whereas groups sharing the same letter are not significantly different, based on one-way ANOVA followed by an uncorrected Fisher's LSD multiple-comparison test ($P < 0.05$).
